# A CRISPR Screen Identifies LAPTM4A and TM9SF Proteins as Glycolipid-Regulating Factors

**DOI:** 10.1016/j.isci.2018.12.039

**Published:** 2019-01-03

**Authors:** Toshiyuki Yamaji, Tsuyoshi Sekizuka, Yuriko Tachida, Chisato Sakuma, Kanta Morimoto, Makoto Kuroda, Kentaro Hanada

**Affiliations:** 1 Department of Biochemistry and Cell Biology, National Institute of Infectious Diseases, 1-23-1 Toyama, Shinjuku-ku, Tokyo 162-8640, Japan; 2 Pathogen Genomics Center, National Institute of Infectious Diseases, 1-23-1 Toyama, Shinjuku-ku, Tokyo 162-8640, Japan; 3 Department of Materials and Applied Chemistry, College of Science and Technology, Nihon University, Chiyoda-ku, Tokyo 101-8308, Japan

**Keywords:** Molecular Biology, Molecular Mechanism of Behavior, Cell Biology, Functional Aspects of Cell Biology

## Abstract

Glycosphingolipids (GSLs) are produced by various GSL-synthesizing enzymes, but post-translational regulation of these enzymes is incompletely understood. To address this knowledge disparity, we focused on biosynthesis of globotriaosylceramide (Gb3), the Shiga toxin (STx) receptor, and performed a genome-wide CRISPR/CAS9 knockout screen in HeLa cells using STx1-mediated cytotoxicity. We identified various genes including sphingolipid-related genes and membrane-trafficking genes. In addition, we found two proteins, LAPTM4A and TM9SF2, for which physiological roles remain elusive. Disruption of either *LAPTM4A* or *TM9SF2* genes reduced Gb3 biosynthesis, resulting in accumulation of its precursor, lactosylceramide. Loss of LAPTM4A decreased endogenous Gb3 synthase activity in a post-transcriptional mechanism, whereas loss of TM9SF2 did not affect Gb3 synthase activity but instead disrupted localization of Gb3 synthase. Furthermore, the Gb3-regulating activity of TM9SF2 was conserved in the TM9SF family. These results provide mechanistic insight into the post-translational regulation of the activity and localization of Gb3 synthase.

## Highlights

• Genome-wide CRISPR knockout screening using Shiga toxin-induced cell death
• Both LAPTM4A and TM9SF2 are required for Gb3 biosynthesis
• Loss of LAPTM4A reduces Gb3 synthase activity post-transcriptionally
• TM9SF2 regulates the subcellular localization of Gb3 synthase

## Graphical Abstract

**Figure:**
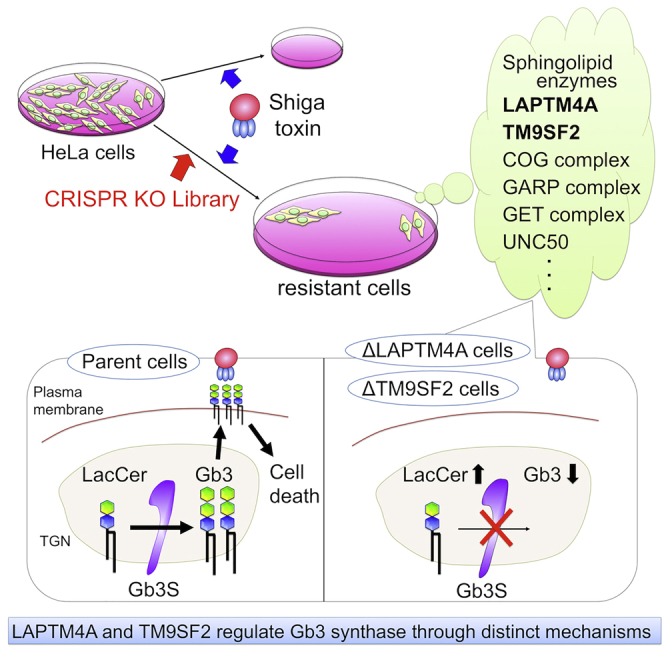

## Introduction

Glycosphingolipids (GSLs) are ubiquitously expressed in animals and are essential for embryonic development (Yamashita et al., 1999). Mammalian cells produce a variety of GSLs, depending on the cell and tissue types. Various physiological roles of GSLs have been identified, including cell adhesion and cell signaling (Hakomori, 2008). In addition, several GSLs are exploited as membrane receptors by toxins and infectious agents. For example, globotriaosylceramide (Gb3) serves as the receptor of Shiga toxin (STx) produced by enterohemorrhagic *Escherichia coli* and *Shigella dysenteriae*, whereas the ganglioside GM1 serves as the receptor of cholera toxin produced by *Vibrio cholerae* (Hanada, 2005). Gb3 also has other biological significance, especially under pathological conditions, including tumor metastasis (Kovbasnjuk et al., 2005) and Fabry diseases, caused by α-galactosidase A deficiency (Clarke, 2007). Loss of Gb3 and the corresponding globo-series GSLs in mice results in higher sensitivity to lipopolysaccharides (Kondo et al., 2013), indicating that the balance of GSLs affects inflammation. Therefore, the regulatory mechanisms of GSL synthesis and degradation are important for understanding various physiological and pathological states.

The overall structure of complex glycan moieties in GSLs is highly diverse. Nevertheless, their core portion is conserved; the hydrophobic moiety of GSLs is commonly composed of ceramides, which are synthesized in the ER. After transport from the ER to the late Golgi complex by the ceramide transport protein CERT (Hanada et al., 2003), ceramide is converted to sphingomyelin, a major phosphosphingolipid in mammals. On the other hand, if ceramide is transported to the early Golgi region through a CERT-independent mechanism, ceramide is converted to glucosylceramide (GlcCer), which is the common precursor of all GSLs, with exception to galactosylceramide and its derivatives (Ichikawa et al., 1996). After traversing across the Golgi membrane, GlcCer is converted to lactosylceramide (LacCer) in the luminal side of the Golgi complex (Kumagai et al., 2010). LacCer is converted to one of several types of trihexosyl ceramides, which in mammals are composed predominately of Gb3 and GM3. Gb3 is synthesized from LacCer by α1,4 galactosyltransferase (hereafter referred to as Gb3 synthase; encoded by the *A4GalT* gene in the human genome), which is mainly localized to the *trans-*Golgi network (TGN) (Kojima et al., 2000, Yamaji and Hanada, 2015, D'Angelo et al., 2013).

Proper glycosylation of GSLs requires not only transcriptional regulation of GSL enzyme genes but also post-translational regulation of these enzymes, including the regulation of enzymatic activities, subcellular distributions, and transports. For example, the conserved oligomeric Golgi (COG) complex maintains Golgi-resident glycan enzymes by retrograde trafficking (Blackburn and Lupashin, 2016), and defects in COG subunits are linked to Congenital Disorders of Glycosylation-type II, a group of inherited metabolic disorders (Zeevaert et al., 2008). However, the mechanisms of post-transcriptional glycosyltransferase regulation in the Golgi and TGN are incompletely understood.

To further elucidate mechanisms of post-transcriptional GSL regulation, we focused on the biosynthesis of the STx receptor Gb3 and performed a genome-wide CRISPR/CAS9 knockout (KO) screen (Shalem et al., 2014, Wang et al., 2014) in HeLa cells using STx1-induced cytotoxicity as our screening criteria. This allowed for unbiased identification of intracellular factors affecting the activity and localization of the Gb3-related enzymes through screening for gene mutations conferring STx resistance. This approach identified two multispanning membrane proteins, lysosomal protein transmembrane 4α (LAPTM4A) and transmembrane 9 superfamily 2 (TM9SF2). Loss of either protein resulted in reduction of Gb3 levels and subsequent STx resistance. Biochemical and cell biological analyses revealed that LAPTM4A and TM9SF2 regulated Gb3 synthase through distinct mechanisms, providing insight into previously unrecognized mechanisms for post-translational regulation of GSL synthases.

## Results

### Identification of Genes Conferring Resistance to STx-Induced Cell Death

**Figure 1:**
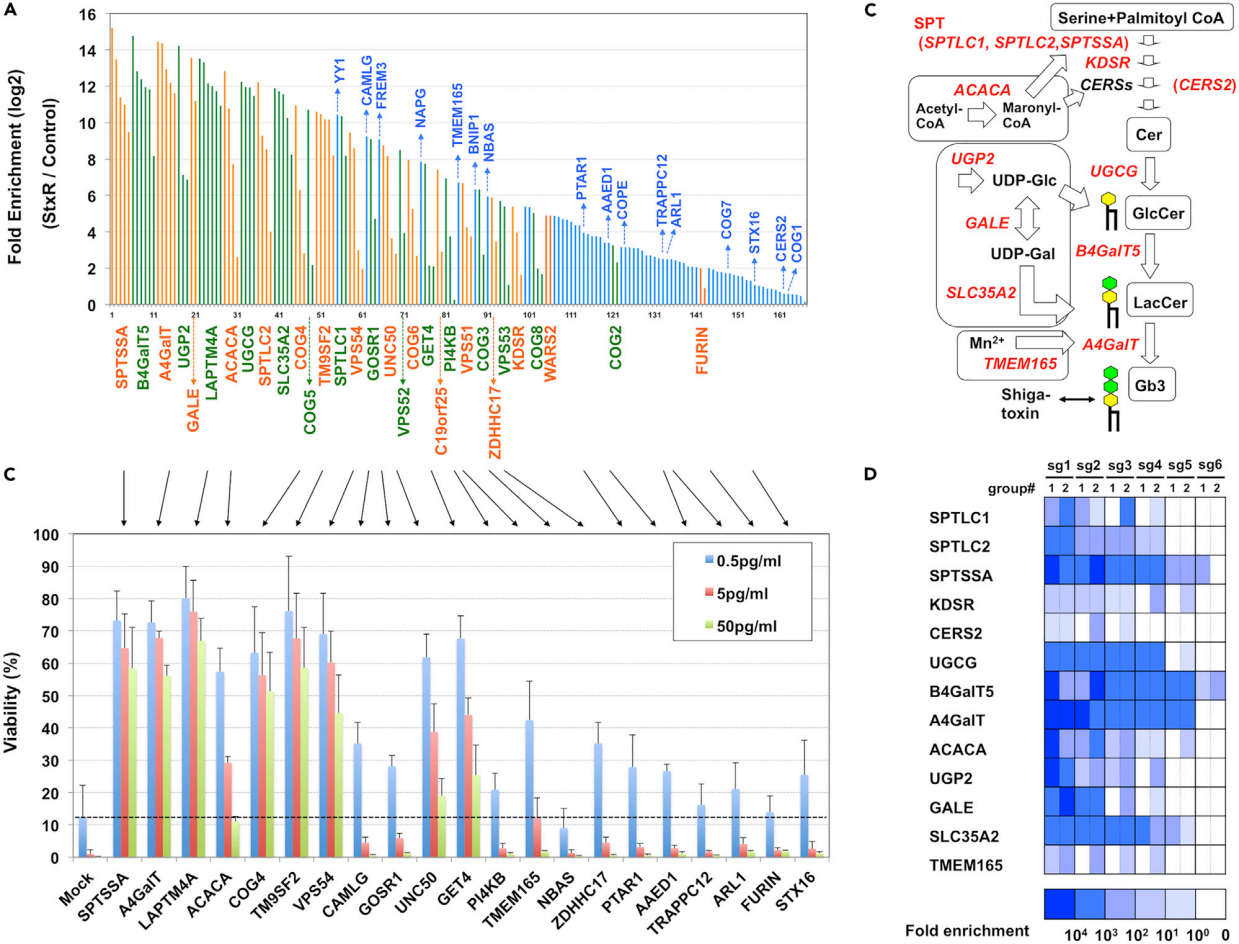
Identification of STx Resistance Genes in a Genome-Wide CRISPR Screen (A) Identification sgRNAs enriched in the screen. Fold enrichment represents the average of two independent experiments. Orange and green bars indicate that multiple sgRNAs were enriched in a gene, whereas blue bars indicate that a single sgRNA was enriched in a gene. The full raw dataset is shown in Data S2. (B) Reproducibility of STx resistance conferred by individual sgRNAs. Each sgRNA was transduced into HeLa cells. Untransfected cells were excluded using puromycin selection, and successfully transfected cells were then treated with STx1 at the indicated concentration. Viability was estimated using an MTT assay and is expressed as the percentage of the MTT value (OD570) in the absence of STx1. Percentage shown is mean percentage ±SD obtained from three independent experiments. Arrows indicate that the sgRNAs shown in Figure 1A correspond to the sgRNAs in this figure. The dotted line indicates the viability of mock-transfected cells treated with 0.5 pg/mL STx1. (C) Gb3 biosynthetic pathway. Genes enriched in the screen are shown in red. (D) Fold enrichment of six sgRNAs in sphingolipid-related genes shown in Figure 1C. Heatmap is representative individual sgRNA enrichment (sg1-6) in two independent experiments (group #1 and 2). See also Figure S1 and Data S1, S2, and S3.

To identify host factors involved in the regulation of Gb3 biosynthesis by exploiting STx sensitivity as an indicator of cellular Gb3 levels, we performed a genome-wide CRISPR/CAS9 KO screen in HeLa cells. We used a lentivirus-based GeCKO v2 pooled library, which is delivered as two half-libraries (A and B) targeting a total of 19,050 human genes with six single guide RNAs (sgRNAs) per gene (Sanjana et al., 2014). Two independent sgRNA-expressing cell libraries (A-1, A-2, B-1, B-2) were prepared by transducing the lentivirus libraries, and cells were then treated with STx1 to assess toxicity. The sgRNAs integrated into the cellular genomes of surviving cells were amplified by PCR and analyzed with high-throughput sequencing. sgRNAs enriched by STx in both independent cell libraries were selected as STx-resistance sgRNA candidates (Figure 1A, the full raw dataset is shown in Data S1 and S2). The candidates included 167 sgRNAs for 97 genes, with 31 genes containing multiple sgRNAs. The enriched gene candidates included nearly all sphingolipid-related genes, including *A4GalT* (Gb3 synthase) and *B4GalT5* (LacCer synthase), and various membrane trafficking genes, including the COG complex (*COG1-8*) involved in intra-Golgi retrograde transport (Blackburn and Lupashin, 2016), the GARP complex (*VPS51-54*) involved in late endosome–TGN retrograde transport (Bonifacino and Hierro, 2011), the GET complex (*GET4, CAMLG*) involved in ER translocation of tail-anchored membrane proteins (Stefanovic and Hegde, 2007), and *UNC50,* which is involved in late endosome-TGN STx retrograde transport, as was recently identified (Selyunin et al., 2017).

For validation of this screen, 21 identified sgRNAs were individually transduced into HeLa cells to identify the effect of these sgRNAs on STx-induced cytotoxicity (Figure 1B). Most sgRNAs conferred resistance to STx. Furthermore, the degrees of resistance and the fold enrichment of each sgRNA (shown in Figure 1A) were highly correlated, indicating the reproducibility of this screening approach. Figure 1C shows the Gb3 biosynthesis pathway. The sgRNAs of all sphingolipid-related enzymes and transporters shown in this pathway were enriched in the screen (Figure 1D). Among these genes, we established KO cell clones of three genes, including serine palmitoyltransferase small subunit A (*SPTSSA*) (Han et al., 2009), acetyl-CoA carboxylase alpha (*ACACA*), and transmembrane protein 165 (*TMEM165*), a Mn^2+^ transporter required for some glycosyltransferases (Potelle et al., 2016), using the CRISPR/CAS system. We confirmed that disruption of these genes reduced or completely inhibited Gb3 biosynthesis and reduced STx binding, which was reversed by the addition of sphingosine to *SPTSSA*-KO cells, addition of palmitate to *ACACA*-KO cells, and TMEM165 cDNA transfection in *TMEM165*-KO cells (Figure S1).

### Inhibition of Gb3 Synthesis in LacCer in *LAPTM4A*- and *TM9SF2*-KO Cells

**Figure 2:**
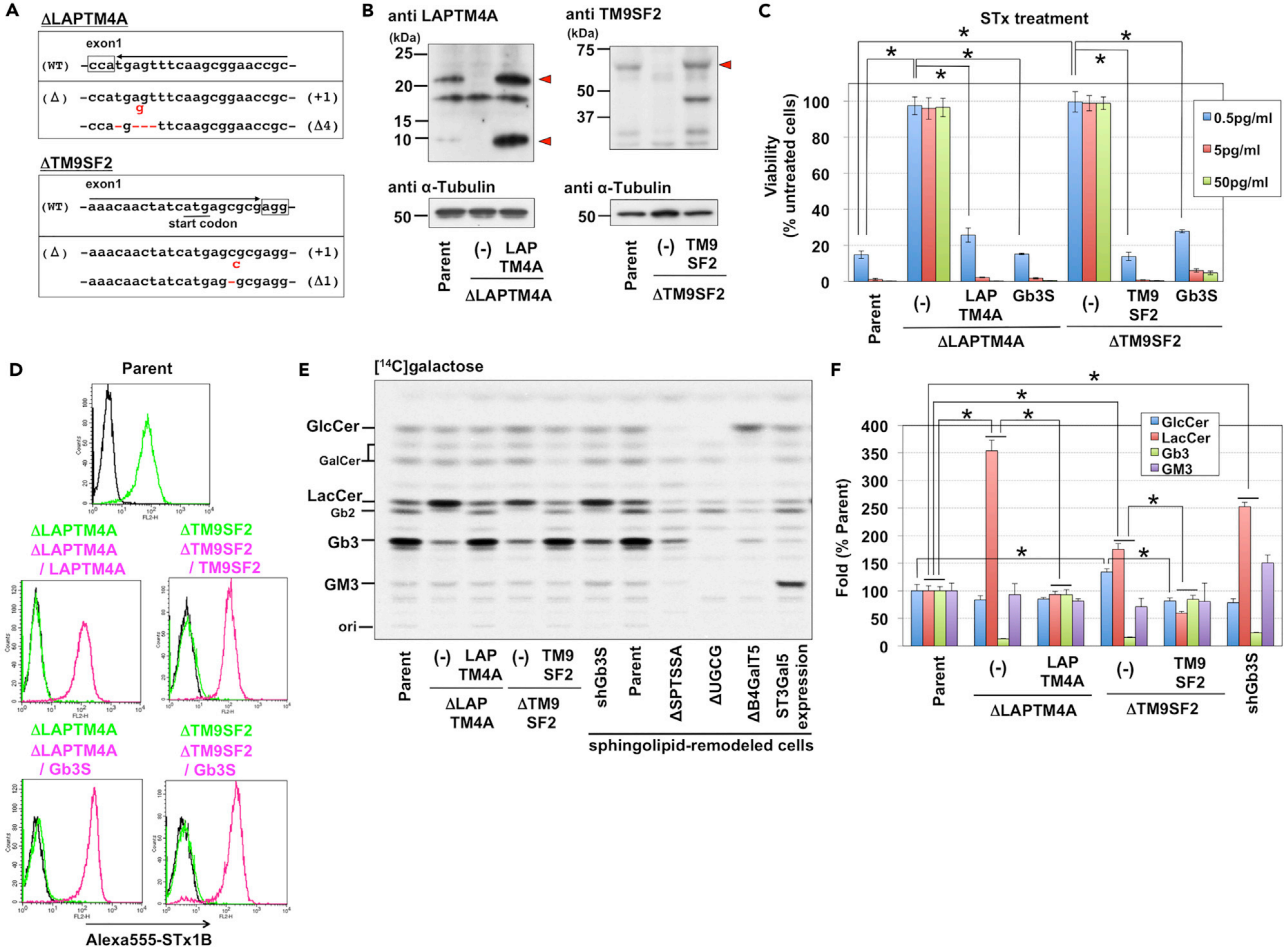
Requirement of LAPTM4A and TM9SF2 for Gb3 Biosynthesis (A) Construction of *LAPTM4A*- and *TM9SF2*-KO HeLa cells. Red letters in sequences are indicative of deletion or insertion mutations, which cause frameshifts shown at the right side of the sequences. Boxes indicate protospacer adjacent motif (PAM) sequences. (B) Western blot analysis of KO cells and cDNA-rescued cells. Parent cells (HeLa mCAT#8), *LAPTM4A*- and *TM9SF2*-KO cells (ΔLAPTM4A and ΔTM9SF2; “-” is indicative of cDNA-unintroduced cells), and corresponding cDNA-reintroduced cells (ΔLAPTM4A/LAPTM4A and ΔTM9SF2/TM9SF2) were analyzed. Triangles are indicative of target proteins. (C) STx sensitivity in KO cells and cDNA-reintroduced cells. Cells shown in B and Gb3S-introduced KO cells were treated with STx1 at the indicated concentrations. Viability was estimated as described in Figure 1B and is expressed as the mean percentage ±SD obtained from three independent experiments. The Bonferroni corrected t test was used for multiple comparisons. *, p < 0.0083. (D) Surface binding of STx on KO cells and corresponding cDNA-rescued cells. Cells shown in C were stained with (yellow-green and magenta lines) or without (black line) Alexa555-labeled STx1 B subunit (Alexa 555-STx1B) and analyzed using FACS. Black and magenta lines indicate staining in KO cells expressing the indicated cDNAs, and yellow-green lines indicate staining in KO cells without introduction of cDNA. (E) GSL metabolic analysis of KO cells and corresponding cDNA-rescued cells. Cells shown in B were labeled with [^14^C]galactose, and labeled lipids treated by mild alkali-catalyzed methanolysis were separated on a TLC plate. To assess lipids accurately, labeled lipids in sphingolipid-remodeled HeLa cells were used as markers, shown at the right side of the samples. UGCG is GlcCer synthase. B4GalT5 is a major LacCer synthase. ST3Gal5 is GM3 synthase. (F) Quantification of labeling experiments shown in E. The relative amount of each [^14^C]galactose-labeled lipid is expressed as the percentage of band intensity in parent cells and is representative of the mean percentage ±SD obtained from three independent experiments. The Bonferroni corrected t test was used for multiple comparisons. *, p < 0.01.

Among the enriched gene candidates shown in Figure 1A, we focused on two multispanning membrane protein genes, *LAPTM4A* and *TM9SF2*, as multiple sgRNAs for these genes were highly enriched in the screen, but their role in STx-induced cell death were as yet unknown. To confirm resistance to STx, KO cell clones of each gene were generated using the CRISPR/CAS system. Sequence analyses demonstrated that coding regions within exon 1 of the respective genes in the *LAPTM4A*- and *TM9SF2*-KO cell clones (ΔLAPTM4A and ΔTM9SF2) were frame-shifted in all alleles (Figure 2A). Furthermore, expression of these proteins was lost in KO cells, as revealed by western blot analysis (Figure 2B). The KO cells were highly resistant to STx-induced cell death (Figure 2C), and cell surface STx binding was also lost (Figure 2D). Introduction of wild-type *LAPTM4A* and *TM9SF2* cDNA into the respective KO cell lines (ΔLAPTM4A/LAPTM4A and ΔTM9SF2/TM9SF2) resulted in full recovery of cell surface Gb3 levels and STx sensitivity (Figures 2C and 2D), verifying that disruptions of *LAPTM4A* and *TM9SF2* were the causative mutations for Gb3-related phenotypes in mutant cells.

To determine if Gb3 biosynthesis was affected by the disruption of *LAPTM4A* and *TM9SF2*, we metabolically labeled lipids with [^14^C]galactose and analyzed the labeled lipids using thin-layer chromatography (TLC) and radioactive imaging (Figures 2E and 2F). Relative to parent cells, *LAPTM4A*- and *TM9SF2*-KO cells produced lower levels of labeled Gb3 (12.9 ± 0.9% in *LAPTM4A*-KO cells and 15.4 ± 0.5% in *TM9SF2*-KO cells) and had higher levels of labeled LacCer, the direct precursor of Gb3 (353.9 ± 19.5% in *LAPTM4A*-KO cells and 175.3 ± 10.4% in *TM9SF2*-KO cells). These labeling patterns in the two mutant cell lines were similar to that of Gb3 synthase knockdown cells (shGb3S). Notably, introduction of wild-type *Gb3 synthase* cDNA into *LAPTM4A*- and *TM9SF2*-KO cells restored both labeled Gb3 levels and STx sensitivity (Figures 2C and 2D). Taken together, these results suggested that LAPTM4A and TM9SF2 were involved in Gb3 synthase-dependent conversion of LacCer to Gb3.

### Loss of LAPTM4A, but Not TM9SF2, Reduced Gb3 Synthase Activity Post-transcriptionally

**Figure 3:**
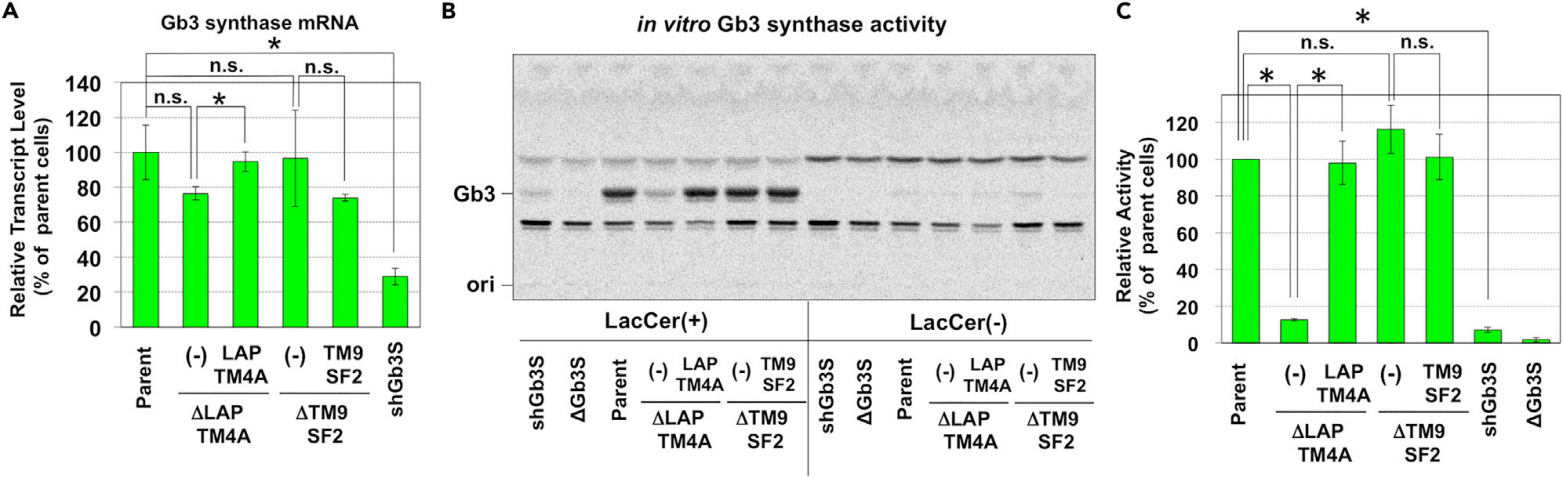
Loss of LAPTM4A, but Not TM9SF2, Reduces Gb3 Synthase Activity Post-transcriptionally (A) Quantitative real-time PCR of Gb3 synthase mRNA. Relative mRNA levels of Gb3 synthase are expressed as the percentage of the value in parent cells and are representative of the mean percentage ±SD obtained from three independent experiments. The Bonferroni corrected t test was used for multiple comparisons. *, p < 0.01. (B) Measurement of Gb3 synthase activity *in vitro*. Cell lysates were incubated with (left side) or without (right side) LacCer in addition to [^3^H]UDP-galactose, and labeled lipids were separated on a high-performance TLC plate. (C) Quantification of labeling experiments shown in B. Relative Gb3 synthase activities are expressed as percentage of the value in control cells and are representative of mean percentage ±SD obtained from three independent experiments. The Bonferroni corrected t test was used for multiple comparisons. *, p < 0.01. See also Figure S2.

Next, the transcriptional level of *Gb3 synthase* was examined in KO cells (Figure 3A). *Gb3 synthase* transcript levels were unchanged in *TM9SF2*-KO cells (lane 4 vs. lanes 1 and 5). *Gb3 synthase* mRNA was modestly decreased in *LAPTM4A*-KO cells (lane 2 vs. lane 3), but this change was not statistically significant compared with control cells (lane 2 vs. lane 1). As a reference, Gb3 synthase-knockdown (shGb3S) cells, which had similar Gb3 levels to *LAPTM4A*-KO cells (Figures 2E and 2F), had a significant reduction of *Gb3 synthase* mRNA (28.9 ± 4.6% compared with the parent cells) (lane 6 vs. lane 1). Taken together, these data suggested that decreased Gb3 levels in *LAPTM4A*- and *TM9SF2*-KO cells were unlikely to be due to transcriptional down-regulation of the *Gb3 synthase* gene. The contribution of post-translational mechanisms, including changes to the stability of Gb3 synthase proteins, was then examined. To this aim, we first attempted to detect endogenous Gb3 synthase proteins using antibodies against Gb3 synthase but were unsuccessful, likely due to low protein abundance (Figure S2). Therefore, we next attempted to measure the enzymatic activity of endogenous Gb3 synthase *in vitro* (Figures 3B and 3C). Intriguingly, Gb3 synthase activity was markedly decreased in *LAPTM4A*-KO cell lysates relative to wild-type. This reduction was recovered to wild-type levels by the introduction of wild-type *LAPTM4A* cDNA. Contrastingly, *TM9SF2* disruption did not affect *in vitro* activity of Gb3 synthase. Taken together, these results indicated that LAPTM4A was involved in the regulation of Gb3 synthase activity or protein abundance, whereas TM9SF2 regulation of Gb3 synthesis was independent of these mechanisms. It should be noted that abundance of exogenously expressed Gb3 synthase was not changed in *LAPTM4A*-KO cells relative to parent cells (Figure S2G).

### Molecular Assessment of LAPTM4A Regulation of Gb3 Synthesis

**Figure 4:**
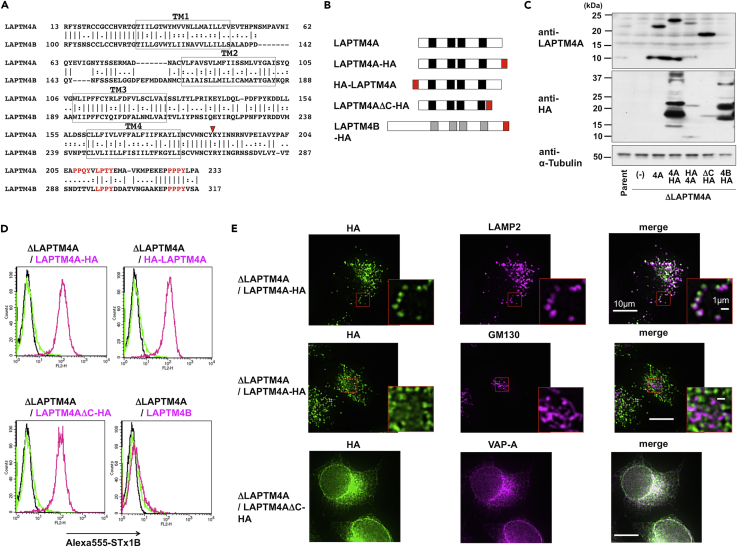
LAPTM4B Does Not Compensate for Loss of LAPTM4A, and the NEDD4-Binding Region of LAPTM4A Is Dispensable for Regulation of Gb3 Biosynthesis (A) Alignment of LAPTM4A and its paralog LAPTM4B. Boxes are indicative of transmembrane domains. Red letters indicate PY motifs (L/PPxY), which can bind to the E3 ubiquitin ligase NEDD4. The triangle is indicative of the C-terminal truncation site (LAPTM4AΔC). (B) Schematics of LAPTM4A, HA-tagged and mutant proteins, and LAPTM4B used in this study. Red boxes indicate HA-tags, and black and gray boxes indicate transmembrane domains. (C) Western blot analysis of stable transfectants expressing the proteins shown in B. Lysates from parent cells, LAPTM4A KO cells (ΔLAPTM4A), and ΔLAPTM4A cells expressing the indicated proteins were used in analysis. Note that the expression level of LAPTM4AΔC-HA was apparently low when anti-HA was used. However, LAPTM4AΔC-HA was detected by anti-LAPTM4A more clearly than anti-HA compared with wild-type LAPTM4A-HA, indicating that LAPTM4AΔC-HA was sufficiently expressed. (D) Surface binding of STx on transfected cells. The indicated cells were stained with (yellow-green and magenta lines) or without (black line) Alexa555-STx1B and analyzed by FACS. Black and magenta lines indicate staining in KO cells expressing the indicated cDNAs, and yellow-green lines indicate staining in KO cells without introduction of cDNA. (E) Intracellular localization of LAPTM4A-HA and LAPTM4AΔC-HA. ΔLAPTM4A/LAPTM4A-HA and ΔLAPTM4A/LAPTM4AΔC-HA cells were stained with anti-HA antibodies and the indicated marker antibodies (anti-LAMP2 [lysosome and late endosome], anti-GM130 [Golgi], anti-VAP-A [ER]). Scale bars, 10 μm and 1 μm. See also Figure S3.

LAPTM4A has four predicted transmembrane domains followed by a C-terminus cytoplasmic tail that contains PY (Leu/Pro-Pro-X-Tyr) motifs for binding of the ubiquitin ligase NEDD4, which is required for lysosomal localization (Figure 4A) (Milkereit and Rotin, 2011). We therefore investigated whether the lysosomal localization of LAPTM4A is required for regulation of Gb3 biosynthesis using a deletion mutant lacking the C-terminal region (LAPTM4AΔC-HA) (Figures 4A and 4B). HA-tagging of LAPTM4A at either the N or C terminus (HA-LAPTM4A or LAPTM4A-HA) had no effect on LAPTM4A regulation of Gb3, and both tagged wild-type proteins recovered STx binding when expressed in *LAPTM4A*-KO cells (Figures 4C and 4D). Intriguingly, deletion of the C-terminal region (LAPTM4AΔC-HA) still maintained Gb3-regulating activity, and the mutant restored defective STx binding in *LAPTM4A*-KO cells (Figures 4C and 4D). Immunostaining analysis with anti-HA revealed that LAPTM4AΔC-HA proteins were mainly localized to the ER (VAP-A), whereas wild-type LAPTM4A-HA and HA-LAPTM4A were mainly localized to lysosomes (LAMP2) and late endosomes (Rab9) (Figures 4E, S3A, and S3B). Taken together, these results suggested that lysosomal and late endosomal localization of LAPTM4A was unlikely to be required for regulation of Gb3 biosynthesis. When these images were carefully observed, we found that LAPTM4A-HA, HA-LAPTM4A, and LAPTM4AΔC-HA were all partially localized to the Golgi apparatus (GM130), suggesting that these proteins may also function in the Golgi (Figures 4E, S3C, and S3D).

**Figure 5:**
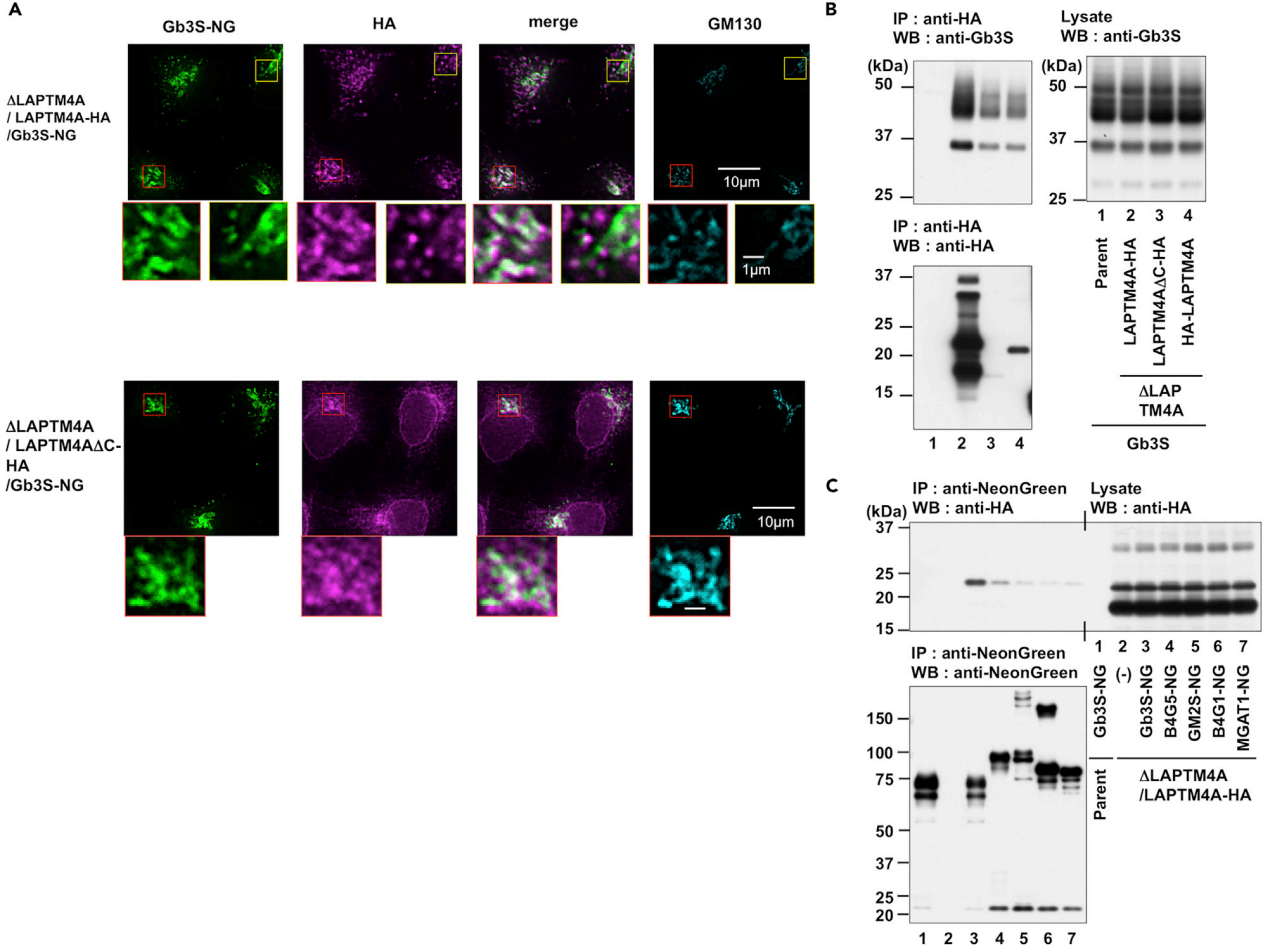
LAPTM4A Interacts with Gb3 Synthase (A) Intracellular localization of LAPTM4A-HA and LAPTM4AΔC-HA in Gb3S-NG-expressing cells. ΔLAPTM4A/LAPTM4A-HA/Gb3S-NG and ΔLAPTM4A/LAPTM4AΔC-HA/Gb3S-NG cells were stained with anti-HA and anti-GM130 antibodies. Scale bars, 10 μm and 1 μm. (B) Co-immunoprecipitation of Gb3 synthase with LAPTM4A. Parent-Gb3S, ΔLAPTM4A/LAPTM4A-HA/Gb3S, ΔLAPTM4A/LAPTM4AΔC-HA/Gb3S, and ΔLAPTM4A/HA-LAPTM4A/Gb3S cells were lysed and immunoprecipitated with anti-HA agarose. Immunoprecipitates (IP) and lysates were subjected to SDS-PAGE and western blot (WB) analyses with the indicated antibodies. (C) Co-immunoprecipitation of LAPTM4A with glycosyltransferases. Parent/Gb3S-NG cells and ΔLAPTM4A/LAPTM4A-HA cells expressing Gb3S-NG, B4GalT5 (B4G5)-NG, GM2 synthase (GM2S)-NG, B4GalT1 (B4G1)-NG, and MGAT1-NG were lysed and immunoprecipitated with anti-NeonGreen magnet beads. Immunoprecipitates (IP) and lysates were subjected to SDS-PAGE and western blot (WB) with the indicated antibodies. See also Figure S4.

Next, to assess intracellular localization of Gb3 synthase, moxNeonGreen fluorescent protein (Costantini et al., 2015, Shaner et al., 2013) was fused to the C-terminus of Gb3 synthase (Gb3S-NG), and fusion proteins were retrovirally expressed in parent cells (Parent/Gb3S-NG), *LAPTM4A*-KO cells (ΔLAPTM4A/Gb3S-NG), and LAPTM4A-rescued cells (ΔLAPTM4A/LAPTM4A-HA/Gb3S-NG and ΔLAPTM4A/LAPTM4AΔC-HA/Gb3S-NG). Gb3S-NG was mainly localized to the Golgi (likely including the TGN) in both parent and *LAPTM4A*-KO cells, although some dispersed punctate structures were observed in a few cell populations. The similar localization patterns of Gb3S-NG suggested that LAPTM4A did not affect localization of Gb3 synthase (Figure S4). In Gb3S-NG-expressing cells, LAPTM4A-HA and LAPTM4AΔC-HA were partially co-localized with Gb3S-NG at the Golgi apparatus (Figure 5A). In particular, in addition to a weak staining of LAPTM4A-HA at the Golgi seen in some cell populations (indicated by the yellow box in Figure 5A) such as Gb3S-NG-unexpressing cells (Figure 4E), strong staining of LAPTM4A-HA at the Golgi was observed in some cell populations, and the staining co-localized with Gb3-NG (red box in Figure 5A). These results prompted us to investigate the interaction between LAPTM4A and Gb3 synthase. Therefore, we performed immunoprecipitation analysis using LAPTM4A and Gb3S-coexpressing cells. When anti-HA-agarose was used, Gb3 synthase co-immunoprecipitated with LAPTM4A-HA, HA-LAPTM4A, and LAPTM4AΔC-HA (Figure 5B). No band was detected when only Gb3S was expressed, suggesting that these interactions were specific. Next, several glycosyltransferase-NG fusion proteins (B4GalT5 (B4G5), GM2 synthase (GM2S), B4GalT1 (B4G1), and MGAT1 in addition to Gb3S) were expressed in ΔLAPTM4A/LAPTM4A-HA cells to examine their interaction with LAPTM4A-HA using anti-NeonGreen beads. LAPTM4A-HA immunoprecipitated with Gb3S-NG much more than with the other tested glycosyltransferase (GT)-NGs, suggesting that LAPTM4A preferentially interacted with Gb3S (Figure 5C). Interestingly, an upper band (≈23kDa), but no lower band (≈18kDa), co-immunoprecipitated with Gb3S-NG. The band of HA-LAPTM4A was observed only at 23 kDa (Figure 5B), which is the same as the upper band of LAPTM4A-HA, suggesting that LAPTM4A-HA in the lower band may have been an N-terminal truncation form. Therefore, LAPTM4A may interact with Gb3S at its N-terminal region.

Human LAPTM4A has 46% amino acid homology with human LAPTM4B, another LAPTM family member involved in tumor progression (Meng et al., 2016) and lysosomal ceramide transport (Blom et al., 2015) (Figure 4A). However, LAPTM4B did not compensate for the reduction of Gb3 due to LAPTM4A deficiency (Figures 4B–4D), indicating that the molecular activity of LAPTM4B differs from that of LAPTM4A.

### Molecular Assessment of TM9SF2 Regulation of the Gb3 Synthesis

**Figure 6:**
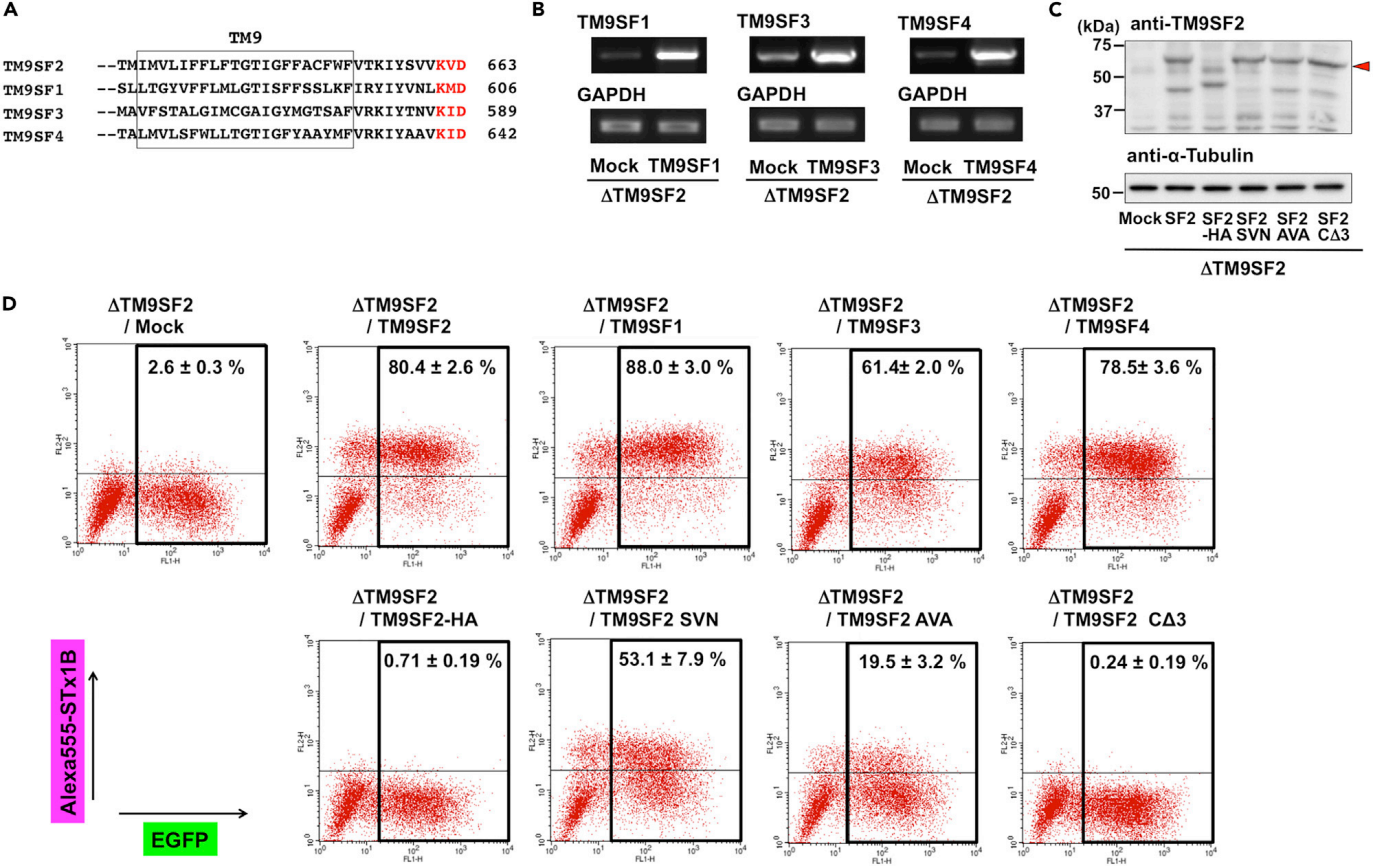
Gb3 Regulation Is Conserved in TM9SF Family Proteins, and the C-terminus of TM9SF2 is Functionally Essential (A) Alignment of C-terminal regions in TM9SF family proteins. Boxes are indicative of transmembrane domains. Red letters are indicative of predicted COPI-binding sites. (B) RT-PCR analysis of *TM9SF1*, *3*, and *4* mRNAs from transient transfectants expressing these TM9SFs. (C) Western blot analysis of transient transfectants expressing HA-tagged TM9SF2 and TM9SF2 mutant proteins. Triangles indicate target proteins. (D) Effects of TM9SF2 family proteins and TM9SF2 mutant proteins on STx binding. cDNAs coding the specified proteins were transiently transfected with EGFP cDNA into *TM9SF2*-KO cells, and cells were stained with Alexa555-STx1B and analyzed by FACS. EGFP-positive cells were gated, and the percentage of STx-binding positive cells is expressed and is representative of the mean percentage ±SD obtained from three independent experiments. See also Figures S5 and S6.

TM9SF2 is a member of the TM9SF family characterized by nine transmembrane domains, with four reported family members (TM9SF1-4) in mammals (Figure 6A) (Chluba-de Tapia et al., 1997, Schimmöller et al., 1998, Lozupone et al., 2009). However, the functional similarity of these proteins is currently unknown. Therefore, we examined whether TM9SF1, 3, or 4 was able to compensate for the loss of TM9SF2 using transient transfection of these cDNAs into *TM9SF2*-KO cells. Expression of TM9SF1–4 was confirmed at the transcriptional level (Figure 6B for TM9SF1, 3, and 4) or at the translation level (Figure 6C for TM9SF2). Fluorescence-activated cell sorting (FACS) analysis revealed that all TM9SFs restored STx binding in *TM9SF2*-KO cells, indicating that regulation of Gb3 synthesis is conserved among TM9SF family members (Figure 6D). The effect of disrupting these TM9SF gene homologs on Gb3 synthesis was then investigated. Transfection of sgRNA targeting to TM9SF2 as well as Gb3S (A4GalT) reduced STx surface binding, whereas sgRNAs targeting other TM9SFs, and a combination of sgRNAs targeting other family members, did not reduce STx binding, although mutations of *TM9SF1*, *3*, and *4* genes occurred as often as that of the *TM9SF2* gene (Figures S5A and S5B). These results indicated that TM9SF2 was the predominant Gb3 regulator among the TM9SF family members, at least in HeLa cells.

TM9SF family proteins have a consensus KxD/E motif (KVD in TM9SF2) at the C terminus, which interacts with the COPI coatomer. In TM9SF family proteins, C-terminal tagging, which masks the terminus, or mutation of the motif, affects interaction with COPI and subcellular localization (Woo et al., 2015). Therefore, we examined whether the C-terminal KxD/E motif of TM9SF2 was required for regulation of Gb3 synthesis. Transfection of C-terminal HA-tagged TM9SF2 (TM9SF2-HA) did not restore STx binding in *TM9SF2*-KO cells (Figure 6D). However, western blot analysis revealed that full-length TM9SF2-HA was not present for unknown reasons (Figure 6C). Therefore, we mutated the C-terminal KxD/E motif (KVD to SVN or AVA) or deleted the motif (CΔ3). These mutations reduced restoration of Gb3 synthesis in *TM9SF2*-KO cells, and CΔ3 resulted in complete loss of this activity, despite expression at levels equivalent to that of wild-type TM9SF2 (Figures 6C and 6D). These results indicated that the C terminus of TM9SF2 is required for the regulation of Gb3 synthesis.

To investigate the interaction between TM9SF2 and Gb3S, an HA-tag was inserted following the putative signal peptide in TM9SF2 (spHA-TM9SF2) (Figure S6A). The spHA-TM9SF2 maintained the ability to restore Gb3 synthesis in *TM9SF2*-KO cells, whereas deletion of the three C-terminal amino acids ablated this ability (Figure S6B). TM9SF2 is known to be localized at the Golgi (Tanaka et al., 2017, Pacheco et al., 2018). However, Golgi localization of spHA-TM9SF2 as well as spHA-TM9SF2 CΔ3 was decreased, although the reason for these differences is unknown (Figure S6C). In this condition, immunoprecipitation analysis using anti-HA agarose beads demonstrated that the high mannose type of Gb3 synthase specifically co-immunoprecipitated with spHA-TM9SF2 and spHA-TM9SF2 CΔ3 but not with GRINA TM4-6-HA, which is a previously reported negative control (Yamaji et al., 2010) (Figure S6D). The selective interaction of high mannose type Gb3 synthase may be due to the localization of spHA-TM9SF2, where only the high mannose type Gb3 synthase was localized. Future studies will clarify whether the interaction with high mannose type Gb3 synthase is specific, or rather is an as yet unidentified artifact. However, taken together, these results suggest that TM9SF2 could interact with Gb3 synthase. Next, spHA-TM9SF2 was transfected into several GT-NG-expressing cell lines to examine their interaction with spHA-TM9SF2. spHA-TM9SF2 immunoprecipitated with Gb3S-NG but did not robustly immunoprecipitate with other GT-NGs, suggesting that TM9SF2 selectively bound Gb3 synthase (Figure S6E).

### TM9SF2 Regulation of Gb3 Synthase Subcellular Localization

As demonstrated earlier, TM9SF2 did not affect the amount or activity of endogenous Gb3 synthase. One hypothesis is that Gb3 synthase may not be able to encounter its substrates in *TM9SF2*-KO cells because of membrane transport defects. Several reports indicated that Gb3 synthase is mainly localized to the TGN rather than to the Golgi cisternae (Yamaji et al., 2010, D'Angelo et al., 2013). Therefore, the intracellular distribution of endogenous TGN46, a TGN marker, was first examined (Figure S7). In parent cells and *LAPTM4A*-KO cells, most TGN46 proteins were merged or aligned with GM130, a cis/medial Golgi marker, whereas in *TM9SF2*-KO cells, more than 30% of cells included dispersed punctate TGN46 staining, which was not merged with GM130. The disruption of TGN46 distribution was restored by expression of TM9SF2. These data suggested that TM9SF2 affected transport of TGN membranes.

**Figure 7:**
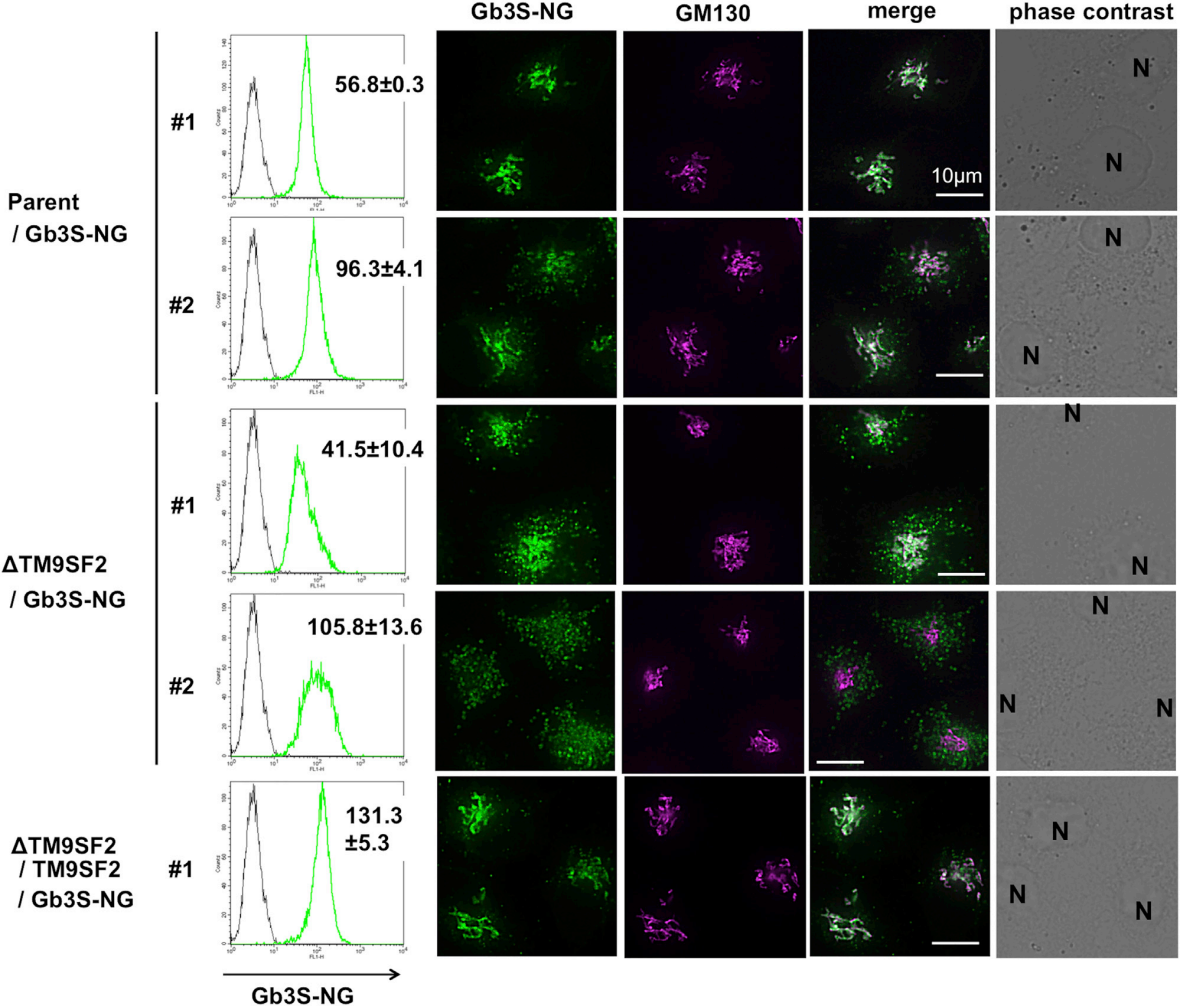
Intracellular Distribution of Exogenously Expressed Gb3S-moxNeonGreen is Disrupted in *TM9SF2*-KO Cells Dispersed punctate structures of Gb3S-moxNeonGreen (NG) in KO cells. Two parent cell clones expressing different levels of Gb3S-NG proteins, two *TM9SF2*-KO cell clones expressing different levels of Gb3S-NG proteins, and a TM9SF2-complemented cell clone expressing higher levels of Gb3S-NG proteins were stained with anti GM130 (Golgi), shown in magenta. Scale bars, 10 μm. Level of exogenously expressed Gb3S-NG in the indicated cell clones was determined by FACS analysis (yellow-green line, the mean ± SD obtained from three independent experiments). See also Figures S4 and S7–S9.

To assess the effect of TM9SF2 on intracellular localization of Gb3 synthase, Gb3S-NG was expressed in *TM9SF2*-KO cells (ΔTM9SF2/Gb3S-NG) and TM9SF2-rescued cells (ΔTM9SF2/TM9SF2/Gb3S-NG) (Figure S4). Western blot analysis revealed that the expression level of Gb3S-NG was similar among these cell lines, and truncation of Gb3S-NG was not observed, suggesting that the fluorescent signal was indicative of full-length Gb3S-NG (Figure S4A). Compared with parent cells, most *TM9SF2*-KO cells contained punctate staining of Gb3S-NG around the Golgi, although the degree of dispersion varied depending on the expression level of Gb3S-NG (Figure S4B). To compare the localization of Gb3 synthase at the same expression level, several clones with different expression levels of Gb3S-NG were isolated. In Gb3S-NG-expressing parent cell clones, most Gb3S-NG proteins were co-localized with or aligned with the Golgi marker GM130 (clone #1), and when the expression level of Gb3S-NG was higher (clone #2, mean fluorescent intensity [MFI] 96.3 compared with MFI 56.8 in clone #1), slight punctate staining appeared around the Golgi (Figure 7). Contrastingly, in Gb3S-NG-expressing *TM9SF2* KO cell clones, more intense punctate staining was observed despite lower expression of Gb3S-NG (clone #1, MFI 41.5), and when the expression of Gb3S-NG was high (clone #2, MFI 105.8), punctate staining was further dispersed. The dispersed punctate staining diminished in Gb3S-NG-expressing TM9SF2-rescued cells, even when the expression of Gb3S-NG was higher in rescued cells (MFI 131.3) than that of Gb3S-NG-expressing KO cell clone #2 (MFI 105.8). These results suggested that TM9SF2 was required for proper Golgi/TGN localization of Gb3 synthase. Unlike TM9SF2, disruption of other TM9SFs (1, 3, and 4) did not affect localization of Gb3S (Figure S5C). On the other hand, intracellular localizations of other GT-NG fusion proteins (B4GalT5, GM2 synthase, B4GalT1, and MGAT1) were also likely perturbed in *TM9SF2*-KO cells (Figure S8). Therefore, the effect of TM9SF2 on transport of Golgi-resident enzymes may be greater than expected, although the effect of glycan metabolism has not yet been examined.

Next, we determined which organelle markers were co-localized with the punctate structures of Gb3S-NG in *TM9SF2*-KO cells (Figure S9). Most punctate dots of Gb3S-NG co-localized with TGN46, a TGN marker protein, but not GM130, a *cis*/*medial*-Golgi marker protein, suggesting that Gb3 synthase was mislocalized together with TGN46. These Gb3S-NG punctate structures were not completely merged with late endosome markers Rab7 and Rab9 and the lysosomal marker LAMP2 but seemed to be in contact with these markers. On the other hand, EEA1, an early endosome marker, did not co-localize with Gb3S-NG punctate structures. Some TGN proteins, including TGN46, are known to be cycled between the TGN, plasma membrane, and endosomes (Ladinsky and Howell, 1992, Pfeffer, 2011). Therefore, as a hypothesis, Gb3 synthase may also cycle between the TGN and endosomes and TM9SF2 may be required for retrograde transport of Gb3 synthase to the TGN/Golgi, which should be clarified in future.

## Discussion

GSL biosynthesis is regulated by a number of factors, but changes in lipid composition have primarily been ascribed to transcriptional regulation of glycosyltransferases. To take an unbiased approach toward identifying novel regulatory mechanisms, we used a genome-wide CRISPR library screen for STx-induced cell death in HeLa cells to demonstrate that various genes, including sphingolipid-related genes and membrane trafficking genes, were comprehensively enriched. In addition, we identified two previously uncharacterized genes, *LAPTM4A* and *TM9SF2*, which were essential for STx binding and subsequent STx cytotoxicity.

Previously, several genome-wide RNAi and CRISPRi screens using STx and cholera toxin, which is another GSL-binding toxin, have been reported (Guimaraes et al., 2011, Gilbert et al., 2014, Selyunin et al., 2017). In genetic screens utilizing toxin-induced cell death, the identified factors may be broadly categorized into two functional groups, comprising genes regulating retrograde trafficking of toxins from the plasma membrane to the ER and cytosol or genes regulating GSL biosynthesis or GSL-synthesizing enzymes. The above-mentioned studies focused primarily on retrograde toxin transport or development of the screening systems. Contrastingly, GSL metabolism has not been well investigated, prompting us to perform the present screen. Some identified genes in our screen were also enriched in prior screens, including *UNC50* (Gilbert et al., 2014, Selyunin et al., 2017) and *TM9SF2* (Gilbert et al., 2014), whereas the GET complex (*GET4* and *CAMLG,* described later) was first observed in this screen.

Just before submission of this manuscript, another group also reported that loss of *LAPTM4A* and *TM9SF2* disrupted STx cell surface binding but did not address the mechanism of action, including the effect of these proteins on Gb3 biosynthesis (Pacheco et al., 2018). We further analyzed the molecular mechanisms for LAPTM4A and TM9SF2 regulation of STx sensitivity using biochemical and cell biology assays. Cumulatively, we demonstrated the following: (1) both LAPTM4A and TM9SF2 were required for Gb3 biosynthesis from LacCer, catalyzed by Gb3 synthase; (2) LAPTM4A and TM9SF2 had differential mechanisms of action, as disruption of LAPTM4A, but not TM9SF2, reduced endogenous Gb3 synthase activity; (3) LAPTM4A and TM9SF2 interacted with Gb3 synthase; (4) ablation of TM9SF2 disrupted the localization of Gb3 synthase; and (5) regulation of Gb3 is conserved in TM9SF family members, and the C-terminus of the proteins is essential for this activity.

The molecular and physiological roles of LAPTM4A remain incompletely understood. Several reports demonstrated that LAPTM4A is mainly localized to lysosomes and late endosomes, at least when it was overexpressed (Cabrita et al., 1999, Hogue et al., 2002, Grabner et al., 2011), which was consistent with our results (Figures 4E and S3). A previous report identified that overexpression of LAPTM4A decreased cell surface localization of OST2 proteins, instead recruiting these proteins to endocytic compartments (Grabner et al., 2011). Another report used genome-wide RNAi screening to identify LAPTM4A as a potential regulator of endosome-to-Golgi retrieval, although a functional analysis was not conducted (Breusegem and Seaman, 2014). LAPTM4A has a cytoplasmic region required for lysosomal sorting through NEDD4 binding (Hogue et al., 2002, Milkereit and Rotin, 2011), but the present study demonstrated that this region is dispensable for LAPTM4A regulation of Gb3 synthesis. Interestingly, C-terminal deletion mutant proteins (LAPTM4AΔC-HA) were mainly localized to the ER (Figure 4E), whereas Gb3 synthase is mainly localized to the TGN. We carefully observed localization of LAPTM4A-HA, HA-LAPTM4A, and LAPTM4AΔC-HA proteins, and all proteins were partially localized to the Golgi apparatus (Figures 4E and S3). Furthermore, LAPTM4A-HA staining at the Golgi was increased by overexpression of Gb3 synthase (Gb3S-NG) (Figure 5A), and LAPTM4A interacted with Gb3 synthase, as demonstrated by immunoprecipitation analysis (Figures 5B and 5C). One potential explanation for these results is that LAPTM4A may cycle between endosome and the Golgi and a small amount of LAPTM4A in the Golgi may be sufficient to localize Gb3 synthase to the TGN/Golgi to regulate its activity or prevent its degradation.

Unfortunately, we were unable to find an anti-Gb3 synthase antibody capable of detecting endogenous Gb3 synthase in western blots and immunofluorescent staining. It is therefore unclear whether decreased Gb3 synthase activity in *LAPTM4A*-KO cells was due to reduction in the amount of Gb3 synthase proteins through synthesis inhibition or degradation or instead was due to inhibition of Gb3 synthase activity without reduction in protein levels. We examined whether loss of LAPTM4A affected the abundance of exogenous Gb3 synthase and found that abundance of exogenous Gb3 synthase was not changed in *LAPTM4A*-KO cells relative to parent cells (Figure S2G). However, this result may not be reflective of endogenous Gb3 synthase regulation, and the ability to detect endogenous Gb3 synthase will better clarify this regulatory mechanism, which will be the topic of future investigations.

LAPTM4B is a paralog of LAPTM4A and has 44% amino acid identity and 63% amino acid similarity. Furthermore, LAPTM4B is also localized to lysosomes and late endosomes through NEDD4 binding. LAPTM4B is known to promote growth of tumor cells and function as a lysosomal ceramide exporter (Blom et al., 2015, Meng et al., 2016). However, there were no prior reports addressing functional similarity between these proteins. We demonstrated in the present study that LAPTM4B did not complement the Gb3 regulatory function of LAPTM4A, suggesting that LAPTM4A has a paralog-specific function that may be related to its multispanning transmembrane domains, which are not conserved in LAPTM4B (Figure 4D). Mutational analyses assessing differential domains between these two proteins will further elucidate the function of LAPTM4A.

Contrary to LAPTM4A, loss of TM9SF2 did not change Gb3 synthase activity *in vitro* despite decreased cellular Gb3 biosynthesis. This suggested that loss of TM9SF2 did not change the protein abundance of Gb3 synthase but instead regulated Gb3 synthase proximity to its substrates, LacCer and UDP-galactose, in intact cells. Gb3S-NG fusion proteins were localized to the Golgi/TGN in parent cells, but in *TM9SF2*-KO cells, Gb3 synthase localization was disrupted, appearing in punctate structures localized with the TGN marker TGN46. Pacheco et al. demonstrated that the localization of both endogenous Gb3S and TGN46 were unchanged in *TM9SF2*-KO cells (Pacheco et al., 2018), which is inconsistent with our results. The reason for this discrepancy is unknown, but it could be due to limitations of endogenous Gb3 synthase. As described earlier, we could not detect a specific signal for endogenous Gb3 synthase by comparison of parent cells with *Gb3S*-KO cells, and it was hard to compare such a low expression level of Gb3 synthase (Figure S2). Instead, we demonstrated that, in addition to the localization of exogenous Gb3 synthase, localization of endogenous TGN46, which was co-localized or aligned with GM130 in parent cells, was also affected in *TM9SF2*-KO cells and that this alteration was restored by overexpression of wild-type TM9SF2 (Figure S7). Taken together with the result that TM9SF2 interacts with Gb3 synthase (Figures S6D and S6E), we suggest that TM9SF2 regulates transport of Gb3 synthase. The punctate Gb3 synthase structures were proximal to late endosomes and lysosomes, suggesting capture by late endosomes/lysosomes (Figure S9). The dynamics of Gb3 synthase transport have not yet been elucidated, but Gb3 synthase may be transported between the TGN and endosomes, and loss of TM9SF2 may affect retrograde transport of Gb3 synthase. Punctate localization of Gb3 synthase was also observed in our prior study, when TMBIM family molecules were overexpressed and Gb3 was decreased (Yamaji et al., 2010). Therefore, perturbed distribution of Gb3 synthase in *TM9SF2*-KO cells may explain the subsequent decrease in Gb3 synthesis, as mislocalization of Gb3 synthase and subsequent decreases of LacCer substrate availability would disrupt Gb3 biosynthesis despite unchanged total abundance of Gb3 synthase.

A recent study demonstrated that TM9SF2 is required for the expression of bifunctional heparan sulfate N-deacetylase/N-sulfotransferase 1 (NDST1), which is involved in heparan sulfate biosynthesis, and that loss of TM9SF2 caused defective heparan sulfate synthesis in a HAP1 haploid cell line (Tanaka et al., 2017). In this case, exogenously expressed NDST1 was decreased in *TM9SF2*-KO HAP1 cells. In contrast, in the present study, loss of TM9SF2 did not change the expression of Gb3S-NG in HeLa cells (Figure S4A). This discrepancy may be due to the difference in enzymes used as substrates, potentially due to differences in cell types.

Previous reports demonstrated that TM9SF2 is primarily localized to the Golgi (Woo et al., 2015, Tanaka et al., 2017). The C-terminal region of TM9SF2 is thought to bind COPI coat proteins, which is important for the localization of TM9SF2 (Woo et al., 2015). Our study demonstrated that the C-terminal region of TM9SF2 was functionally important, as HA-tagging the C-terminus or targeted deletion or mutation of the last three amino acids resulted in loss of the Gb3 regulatory activity of TM9SF2. COPI vesicles are important for intra-Golgi and Golgi-ER retrograde transport (Lee et al., 2004, Yang et al., 2011). Consistent with this mechanism, COPE-targeting sgRNA, which codes the COPI coatomer subunit ɛ, was also enriched in this screen, although the fold enrichment was low (Figure 1A). However, this also supports the notion that retrograde transport of TM9SF2 is important for its function. The GSL biosynthesis pattern in *TM9SF2*-KO cells differed slightly from that of *LAPTM4A*-KO cells. In *LAPTM4A*-KO cells, the reduction of Gb3 corresponded with increased levels of its direct precursor LacCer, suggesting that LAPTM4A specifically affected Gb3 synthase. In contrast, *TM9SF2*-KO cells had only a modest increase in LacCer, and GlcCer was also modestly increased (Figures 2E and 2F). This suggests that TM9SF2 may affect not only Gb3 synthase but also other glycosyltransferases. Disturbed localization of various glycosyltransferases in *TM9SF2*-KO cells may account for the observed phenotype, which should be addressed in future studies (Figure S8).

TM9SF2 has three paralogs, TM9SF1 (31% amino acid identity and 49% amino acid similarity), TM9SF3 (31%, 49%), and TM9SF4 (44%, 62%). The respective functions of each TM9SF family member have been previously reported (Bergeret et al., 2008, He et al., 2009, Oo et al., 2014), but conserved activity among family members has not been extensively investigated. The present study demonstrated that all TM9SF family proteins potentially had the same regulatory activity of Gb3 biosynthesis, although the amino acid identity is only 31%. Detailed analyses of conserved amino acids and functional domains among family members will contribute to understanding the molecular machinery of the TM9SF family. On the other hand, disruption of TM9SF1, 3, and 4 did not affect synthesis of Gb3 and localization of Gb3 synthase. This is consistent with the result that only TM9SF2 was enriched in this screening. The quantitative subcellular proteomic analysis referenced earlier demonstrated that all TM9SF family proteins are expressed at similar levels in HeLa cells (Itzhak et al., 2016). Therefore, the Gb3-regulating activity of TM9SF1, 3, and 4 may be less than that of TM9SF2 at endogenous expression levels.

In addition to sphingolipid enzyme genes, various membrane trafficking genes, especially those involved in retrograde transport, were enriched in the screen. Some of these genes, including COG complex, GARP complex, *UNC50*, and *PTAR1*, a putative prenyltransferase for small G proteins, were also enriched in several other genome-wide screens related to proteoglycans (Rift Valley fever virus [Riblett et al., 2015] and Chikungunya virus [Tanaka et al., 2017]), glycoproteins (Ricin [Bassik et al., 2013] and Lassa virus [Jae et al., 2013, Jae et al., 2014]), and glycolipids (cholera toxin [Gilbert et al., 2014] as well as STx [Selyunin et al., 2017]). Previous studies have identified that the COG complex and GARP complex are involved in toxin trafficking and that depletion of these proteins compromises trafficking (Zolov and Lupashin, 2005, Bailey Blackburn et al., 2016, Pérez-Victoria et al., 2008). Recent screening studies indicate that these membrane trafficking genes more generally affect glycosylation, likely through retrograde trafficking defects of glycosyltransferases, which may overlap with retrograde trafficking of toxins. Loss of the GET complex leads to mislocalization of tail-anchored proteins, including syntaxins involved in retrograde transport (Norlin et al., 2016), which may be why the GET complex was isolated in this screen. The genes identified in this screen have not been fully analyzed, and comparison of GSL biosynthesis in KO or knockdown cells will clarify the functional interactions between genes, and the relationship between these factors and glycosylation, which is a subject of future investigations.

During the second revision process of this manuscript, Tian S. et al. published a similar work demonstrating that disruption of LAPTM4A and TM9SF2 reduced Gb3 (Tian et al., 2018). The authors demonstrated that loss of LAPTM4A did not change the expression level of endogenous Gb3 synthase. Together with our data that loss of LAPTM4A reduced Gb3 synthase activity *in vitro*, LAPTM4A may regulate Gb3 synthase activity, rather than reduce the amount of Gb3 synthase.

In summary, we performed a genome-wide loss-of-function screen using a CRISPR library to identify genes that conferred resistance to STx1. Among the enriched genes, we identified two factors, TM9SF2 and LAPTM4A, which post-transcriptionally regulated Gb3 synthase through differential mechanisms. Furthermore, the Gb3-regulating activity of TM9SF2 is conserved among other TM9SF family proteins, although TM9SF2 is the predominant Gb3 regulator among the family proteins in HeLa cells. These results provide mechanistic insight into post-translational regulation of Gb3 synthase activity and localization.

### Limitations of the Study

In this study, we found that loss of LAPTM4A reduced Gb3 synthase activity. Therefore, measurement of endogenous Gb3S protein level in *LAPTM4A* KO cells is important to understand the function of LAPTM4A. We attempted to detect endogenous Gb3 synthase proteins using antibodies against Gb3 synthase. However, unfortunately, we were unable to find an anti-Gb3 synthase antibody capable of detecting endogenous Gb3 synthase in western blots and immunofluorescent staining using parent cells, *Gb3S*-KO cells, and Gb3S-overexpressing cells, although one of three commercially available antibodies clearly detected exogenously expressed Gb3 synthase in both methods (Figures S2A–S2D). In a recent quantitative subcellular proteomic analysis of Gb3 synthase localization, endogenous Gb3 synthase was still below the detection limit in HeLa cells, although approximately 9,000 proteins were detected, including glucosylceramide synthase (UGCG) and lactosylceramide synthase (B4GalT5) (Itzhak et al., 2016). We also demonstrated that the expression level of endogenous Gb3 synthase was less than 1% of exogenously expressed Gb3 synthase (Figure S2C) and HA-tagged Gb3S at an endogenous expression level was only slightly detected when Gb3S-HA was immunoprecipitated, in an analysis using HA-tag knock-in cells (Figures S2E and S2F). These results indicate that the expression level of endogenous Gb3 synthase is quite low. Most overexpressed Gb3 synthase proteins are glycosylated with either high-mannose type or complex type N-glycans in HeLa cells (Figure S2), and it is likely important to distinguish between the two glycosylation types of Gb3 synthase in considering the dynamics of Gb3 synthase (Yamaji et al., 2010). Therefore, the ability to detect both types of endogenous Gb3 synthase quantitatively will better clarify this regulatory mechanism, which will be the topic of future investigations.

## Methods

All methods can be found in the accompanying Transparent Methods supplemental file.

## Data and Software Availability

The sgRNA data reported in this study have been deposited to the NCBI GEO and are available under accession number GEO: GSE116730.

## Supplemental Information

Document S1. Transparent Methods and Figures S1–S9

Data S1. Read Count, Normalization and Statistical Analysis of sgRNA in GeCKO Version 2 Library, Related to Figure 1

Data S2. Summary of Enriched Genes in STx-Resistant Cells, Related to Figure 1

Data S3. Gene List of Library Set A/B Statistically Significant in STx-Resistant Cells, Related to Figure 1
