# Supplementary material for: A CRISPR Screen Identifies LAPTM4A and TM9SF Proteins as Glycolipid-Regulating Factors
Source: iScience. 2019 Jan 3;11:409–24. doi: 10.1016/j.isci.2018.12.039 (PMC6348303; doi:10.1016/j.isci.2018.12.039)
Supplement: Document S1. Transparent Methods and Figures S1–S9 [file mmc1.pdf]

ISCI, Volume *11*

Supplemental Information

A CRISPR Screen Identifies LAPTM4A and TM9SF Proteins as Glycolipid-Regulating Factors

Toshiyuki Yamaji, Tsuyoshi Sekizuka, Yuriko Tachida, Chisato Sakuma, Kanta Morimoto, Makoto Kuroda, and Kentaro Hanada

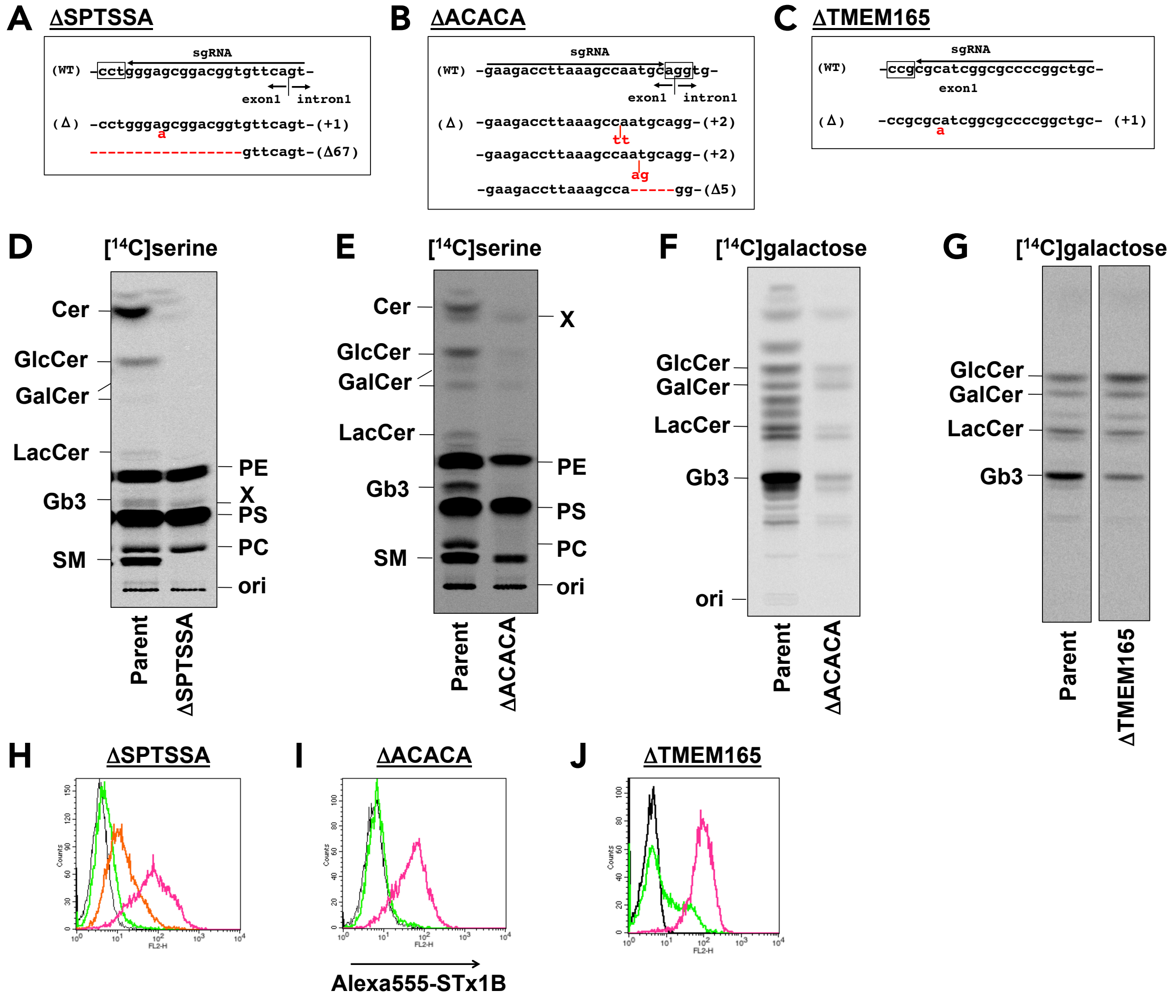

Figure S1. Verification of Gb3 Reduction in Sphingolipid-Related Gene KO Cells, Related to Figure 1.

(A-C) Construction of *SPTSSA*- (A), *ACACA*- (B), and *TMEM165*- (C) KO HeLa cells. Red letters in sequences indicate deletion or insertion mutations, which cause frameshifts shown at the right side of the sequences. Boxes are indicative of protospacer adjacent motif (PAM) sequences.

(D-G) GSL metabolic analysis in sphingolipid-related KO cells. Cells shown in A-C were labeled with [^{14}C]serine (D, E), or [^{14}C]galactose (F, G), and labeled lipids treated with (G) or without (D-F) mild alkali-catalyzed methanolysis were separated on a TLC plate.

(H-J) Surface binding of STx on KO cells. Indicated cells were stained with (yellow-green, orange and magenta lines) or without (black line) Alexa555-STx1B and analyzed by FACS. (H) Yellow-green line indicates staining in KO cells without any other treatment, and the effect of sphingosine addition on STx binding is indicated by the orange line (2μM) and the magenta line (7.5μM). The addition of sphingosine compensates for defective sphingolipid biosynthesis through the salvage pathway. (I) The yellow-green line indicates staining in KO cells without any other treatment, and the effect of palmitate addition on STx binding is indicated by the magenta line (100μM). The addition of palmitate compensates for defective fatty acid biosynthesis. (J) The yellow-green line indicates staining in KO cells without any introduction of cDNA, and magenta line indicates staining in KO cells expressing TMEM165 cDNAs.

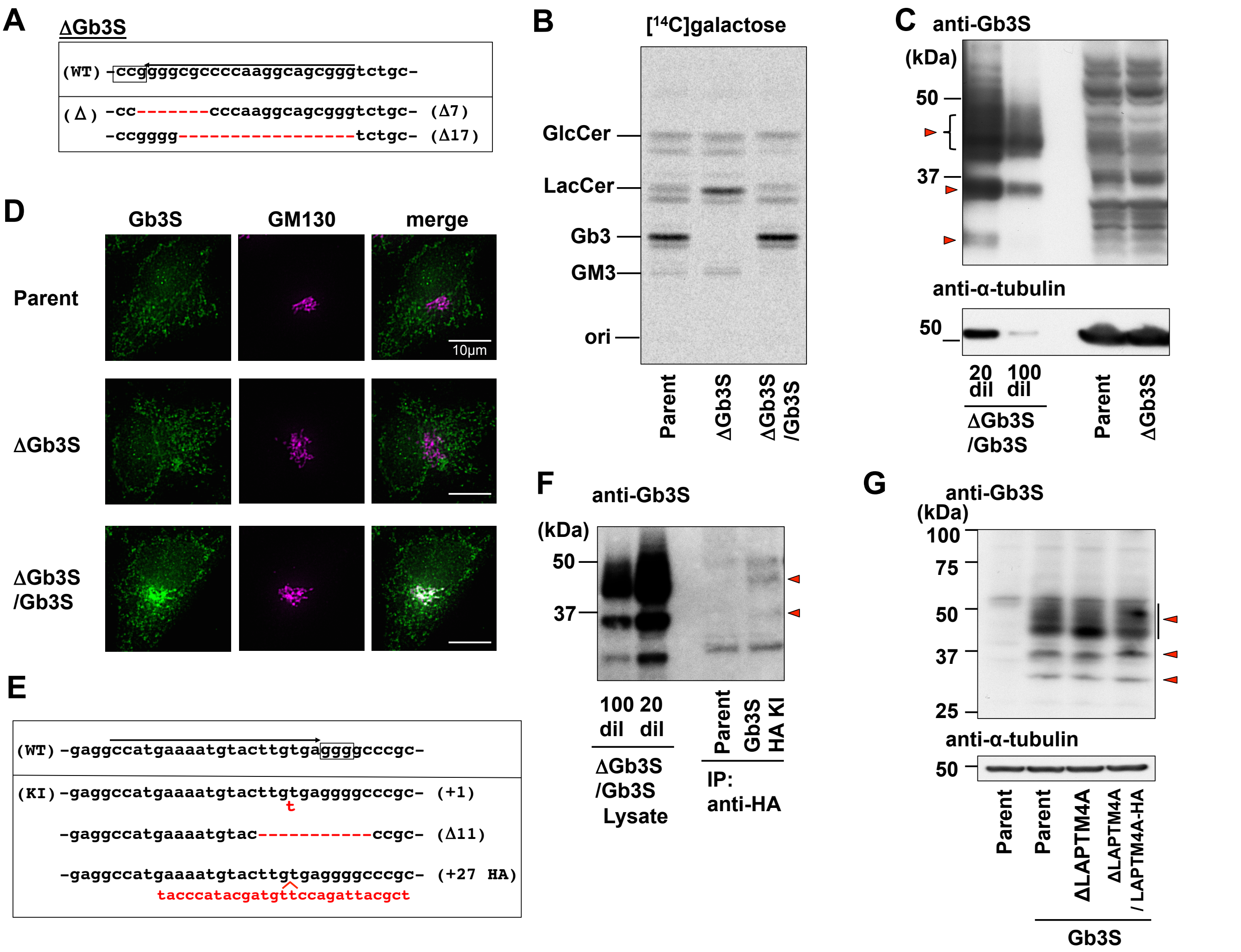

Figure S2. Endogenous Gb3 Synthase Cannot Be Detected Due to Low Expression, Related to Figure 3.

(A) Construction of *Gb3S*-KO HeLa cells. Red letters in sequences indicate deletion mutations, which cause frameshifts shown at the right side of the sequences. Boxes are indicative of PAM sequences.

(B) GSL metabolic analysis in Gb3S-KO cells. Cells were labeled with [^{14}C]galactose, and the labeled lipids were separated on a TLC plate.

(C) Western blot analysis of Gb3S proteins. Parent cells, *Gb3S*-KO cells (ΔGb3S), and Gb3S cDNA-reintroduced cells (ΔGb3S/Gb3S), were analyzed. Lysates of ΔGb3S/Gb3S were loaded at 20- and 100-fold dilutions. Triangles are indicative of Gb3S proteins (complex type, high mannose type, and non-glycosylation type from the top (Yamaji et. al., 2010)). Note that expression level of endogenous Gb3S proteins was lower than that of exogenous Gb3S at 100-fold dilution.

(D) Immunofluorescence analysis of Gb3S proteins. Parent cells, ΔGb3S cells, and ΔGb3S/Gb3Scells were stained with anti-Gb3S antibodies and anti-GM130 (Golgi). Scale bars, 10 μm and 1 μm. Note that endogenous Gb3S was undetected in this condition.

(E) Construction of *Gb3S*-HA knock-in HeLa cells (Gb3S-HA KI). The HA tag sequence was inserted to one of three *Gb3S* gene alleles at the C-terminal side.

(F) Western blot analysis of Gb3S-HA proteins. Lysates of parent cells and Gb3S-HA KI cells were immunoprecipitated with anti-HA agarose. The immunoprecipitates and lysate of ΔGb3S/Gb3S were loaded. Triangles are indicative of Gb3S-HA proteins.

(G) Levels of exogenously expressed Gb3S in parent cells, ΔLAPTM4A cells, and ΔLAPTM4A/LAPTM4A cells. Retrovirally expressed Gb3S proteins in the indicated cells were analyzed by western blotting using anti-Gb3 synthase antibodies. Triangles are indicative of Gb3S proteins.

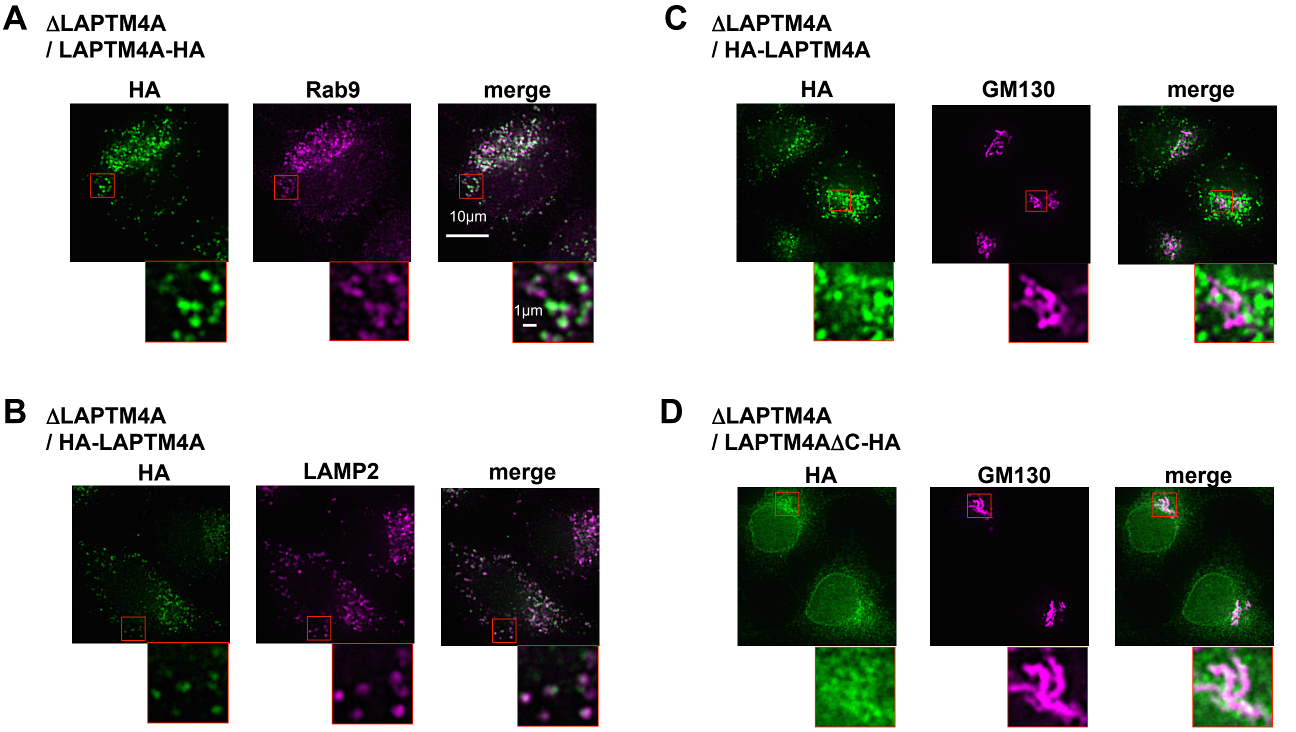

Figure S3. Intracellular Localization of LAPTM4A-HA, HA-LAPTM4A, and LAPTM4AΔC-HA, Related to Figure 4.

ΔLAPTM4A/LAPTM4A-HA, ΔLAPTM4A/HA-LAPTM4A and ΔLAPTM4A/LAPTM4AΔC-HA cells were stained with anti-HA antibodies and the indicated marker antibodies (anti-LAMP2 (lysosome and late endosome), anti-Rab9 (late endosome), anti-GM130 (Golgi)). Scale bars, 10 μm and 1 μm.

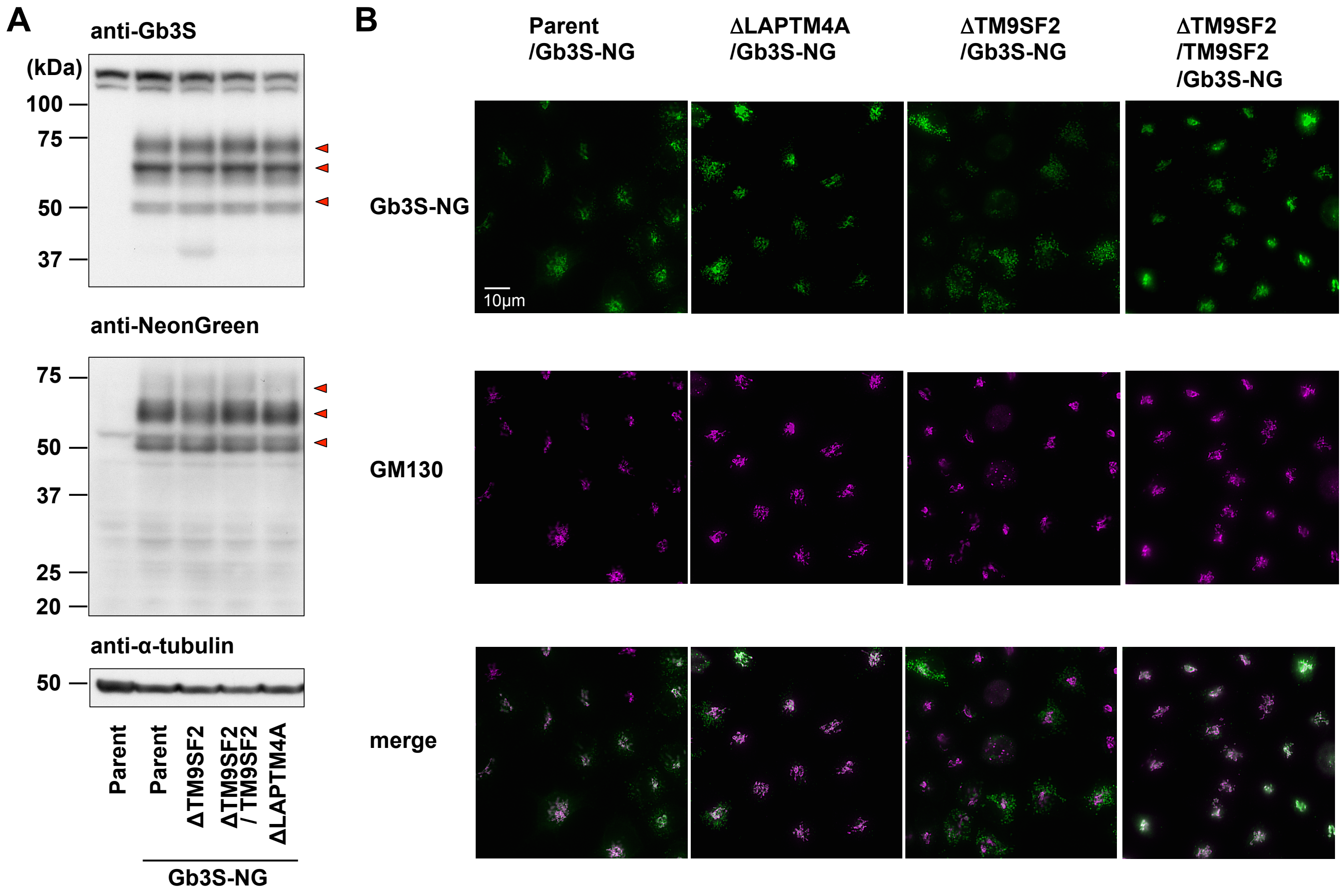

Figure S4. Expression of Gb3S-moxNeonGreen, Related to Figure 5 and 7.

(A) Western blot analysis of Gb3S-moxNeonGreen (NG) proteins. Gb3S-NG was retrovirally expressed in parent cells, *TM9SF2*-KO cells, TM9SF2-rescued cells, and *LAPTM4A*-KO cells. Expression of Gb3S-NG was examined using anti-Gb3S and anti-NeonGreen. Triangles are indicative of Gb3S-NG proteins.

(B) Intracellular localization of Gb3S-NG. Parent/Gb3S-NG cells, ΔTM9SF2/Gb3S-NG cells, ΔTM9SF2/TM9SF2/Gb3S-NG cells, and ΔLAPTM4A/Gb3S-NG cells were stained with anti-GM130 antibodies. Scale bars, 10 μm.

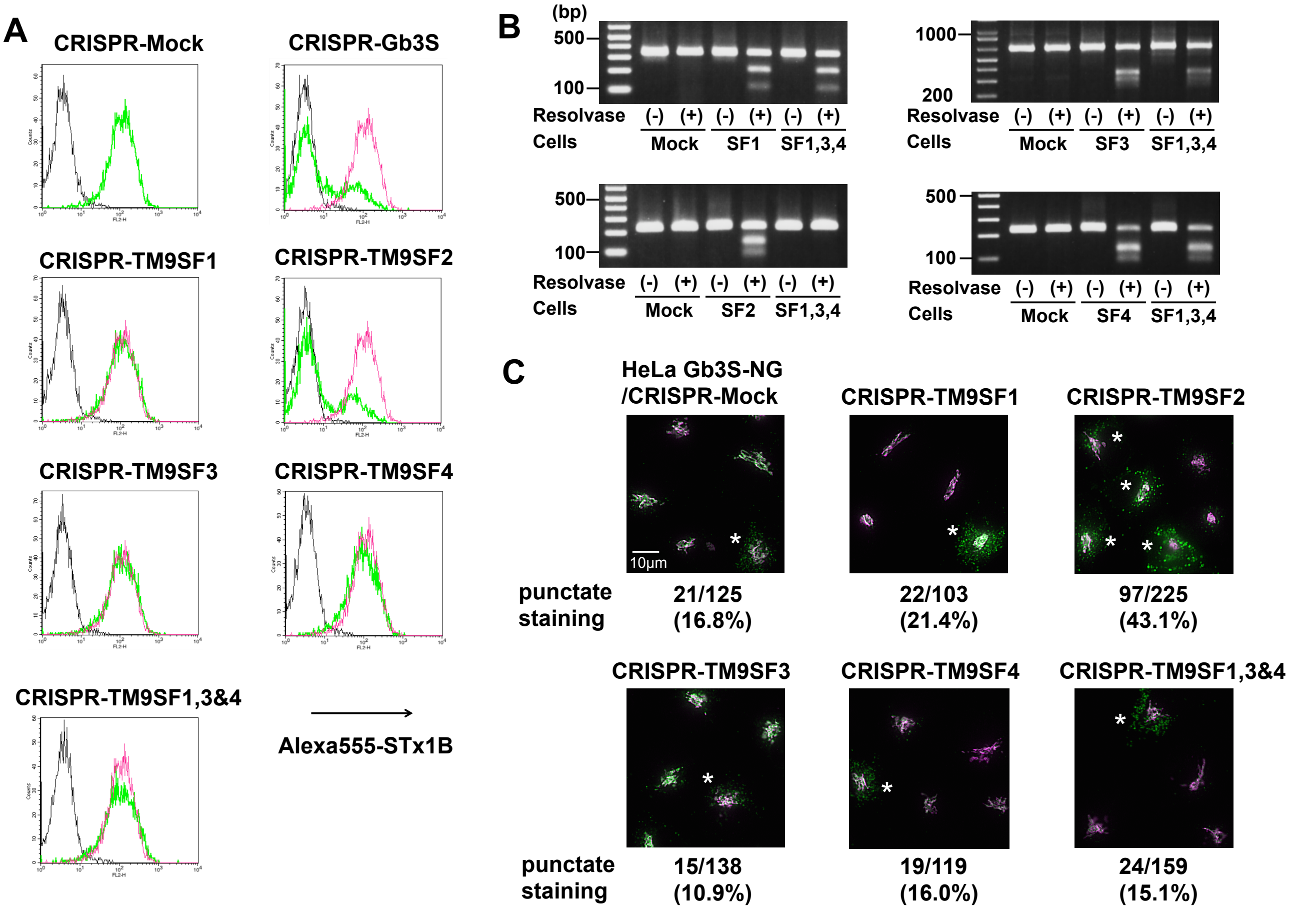

Figure S5. Disruption of Other TM9SF family Genes does not Affect Gb3 Metabolism, Related to Figure 6.

(A) Surface binding of STx on cells treated with sgRNAs targeting TM9SF family members. HeLa cells were treated with sgRNAs targeting to TM9SF1–4, a mixture of TM9SF1, 3, & 4 and A4GalT as well as mock and stained with (yellow-green and magenta lines) or without (black line) Alexa555-STx1B and analyzed using FACS. Magenta lines indicate staining in mock-treated cells as the upper left histogram.

(B) Mutation analysis of sgRNA-treated cells. Genomic PCR fragments containing mutation sites from the cells described in (A) were digested with Resolvase. Cleavage of fragments reflects the degree of mutations.

(C) Intracellular localization of Gb3S-NG in sgRNA-treated cells. Parent/Gb3S#IB1 cells were treated with sgRNAs targeting to TM9SF1–4 and a mixture of TM9SF1, 3, & 4 as well as mock vector. Asterisks are indicative of dispersed punctate staining of Gb3S-NG. Cells were stained with anti-GM130 antibodies. Scale bars, 10 μm.

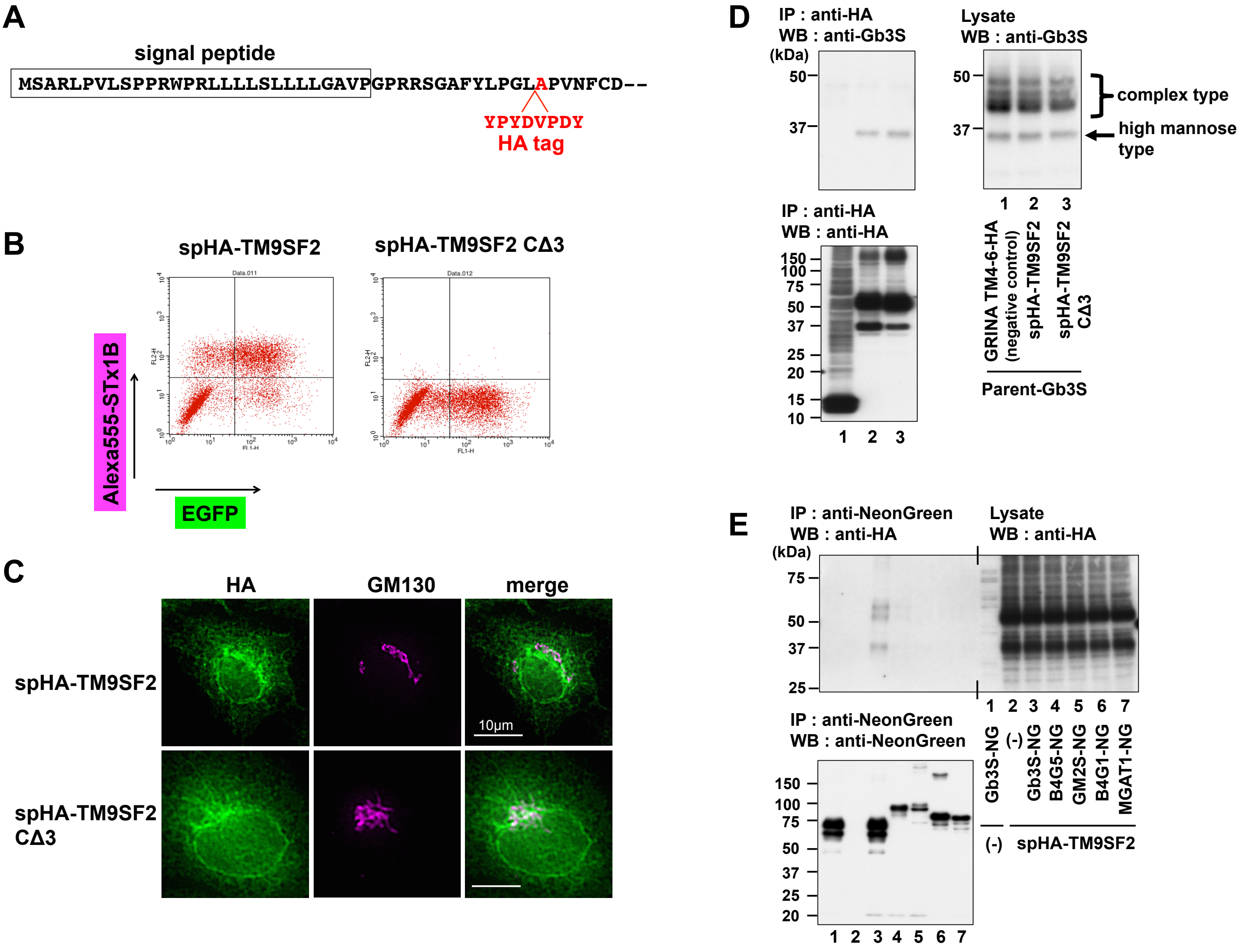

Figure S6. TM9SF2 Interacts with Gb3 Synthase, Related to Figure 6.

(A) Construction of HA-tagged TM9SF2 (spHA-TM9SF2). The HA tag sequence was inserted following the signal peptide.

(B) Effects of spHA-TM9SF2 proteins on STx binding. cDNAs coding spHA-TM9SF2 and spHA-TM9SF2 CΔ3 proteins were transiently transfected with EGFP cDNA into *TM9SF2*-KO cells, and cells were stained with Alexa555-STx1B and analyzed by FACS.

(C) Intracellular localization of spHA-TM9SF2. spHA-TM9SF2 and spHA-TM9SF2 CΔ3 plasmid were transiently transfected to HeLa cells (parent cells), and cells were stained with anti-HA and anti-GM130 antibodies. Scale bars, 10 μm.

(D) Co-immunoprecipitation of Gb3 synthase with spHA-TM9SF2. Plasmids encoding GRINA TM4-6-HA, spHA-TM9SF2, and spHA-TM9SF2 CΔ3 were transiently transfected into parent cells. Cells were lysed and immunoprecipitated with anti-HA agarose. Immunoprecipitates (IP) and lysates were subjected to SDS-PAGE and Western blot (WB) with the indicated antibodies.

(E) Co-immunoprecipitation of spHA-TM9SF2 with various glycosyltransferases. spHA-TM9SF2 plasmid was transfected into parent cells expressing Gb3S-NG, B4GalT5 (B4G5)-NG, GM2 synthase (GM2S)-NG, B4GalT1 (B4G1)-NG, and MGAT1-NG. Cells were lysed and immunoprecipitated with anti-NeonGreen magnetic beads. Immunoprecipitates (IP) and lysates were subjected to SDS-PAGE and Western blot (WB) with the indicated antibodies.

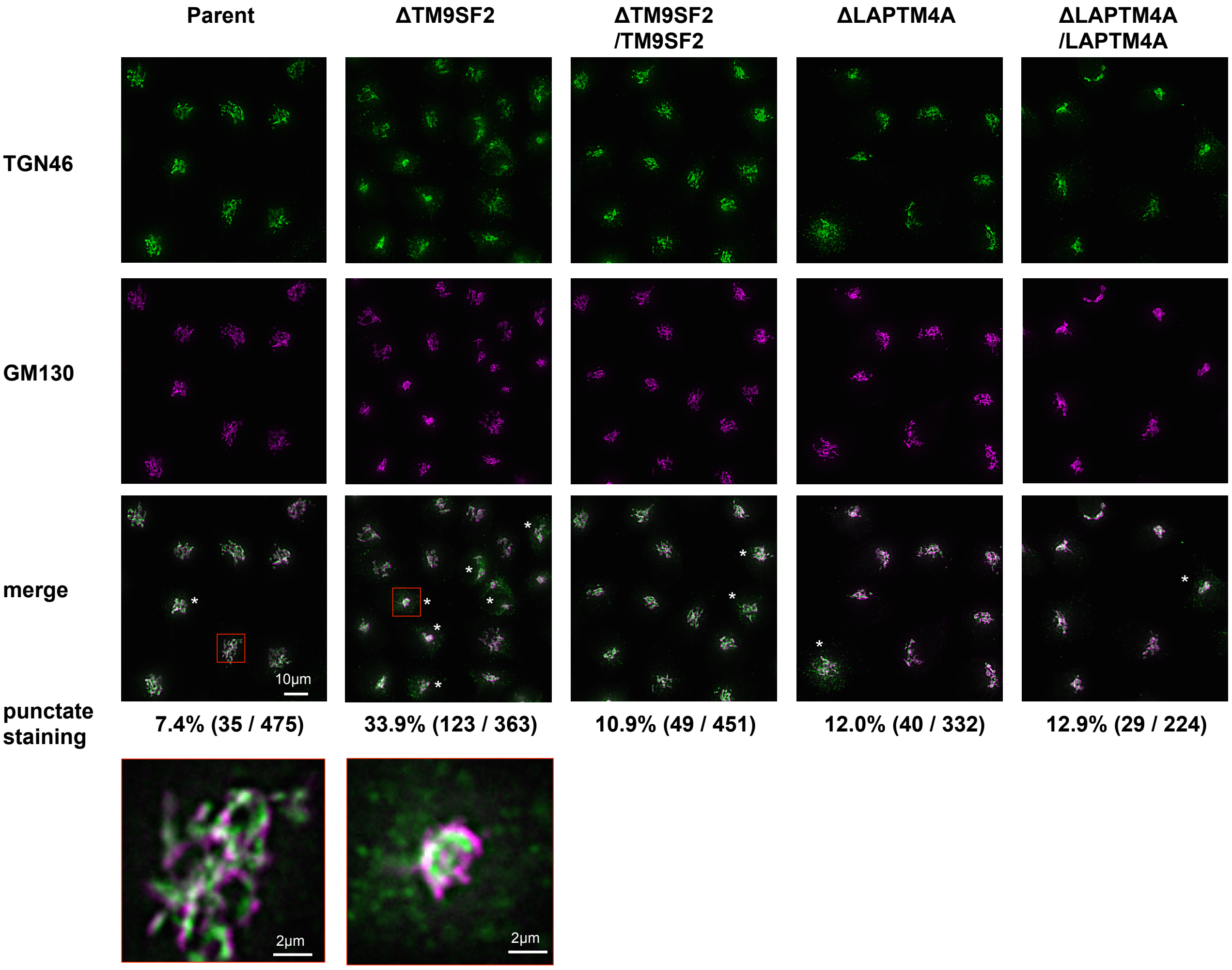

Figure S7. Disruption of TM9SF2 Perturbs Localization of TGN46, Related to Figure 7.

Parent cells, ΔTM9SF2 cells, ΔTM9SF2/TM9SF2 cells, ΔLAPTM4A cells, and ΔLAPTM4A/LAPTM4A cells were stained with anti-GM130 (cis/medial Golgi) and anti-TGN46 (TGN) antibodies. Asterisks are indicative of dispersed punctate TGN46 staining. Scale bars, 10 μm and 2 μm.

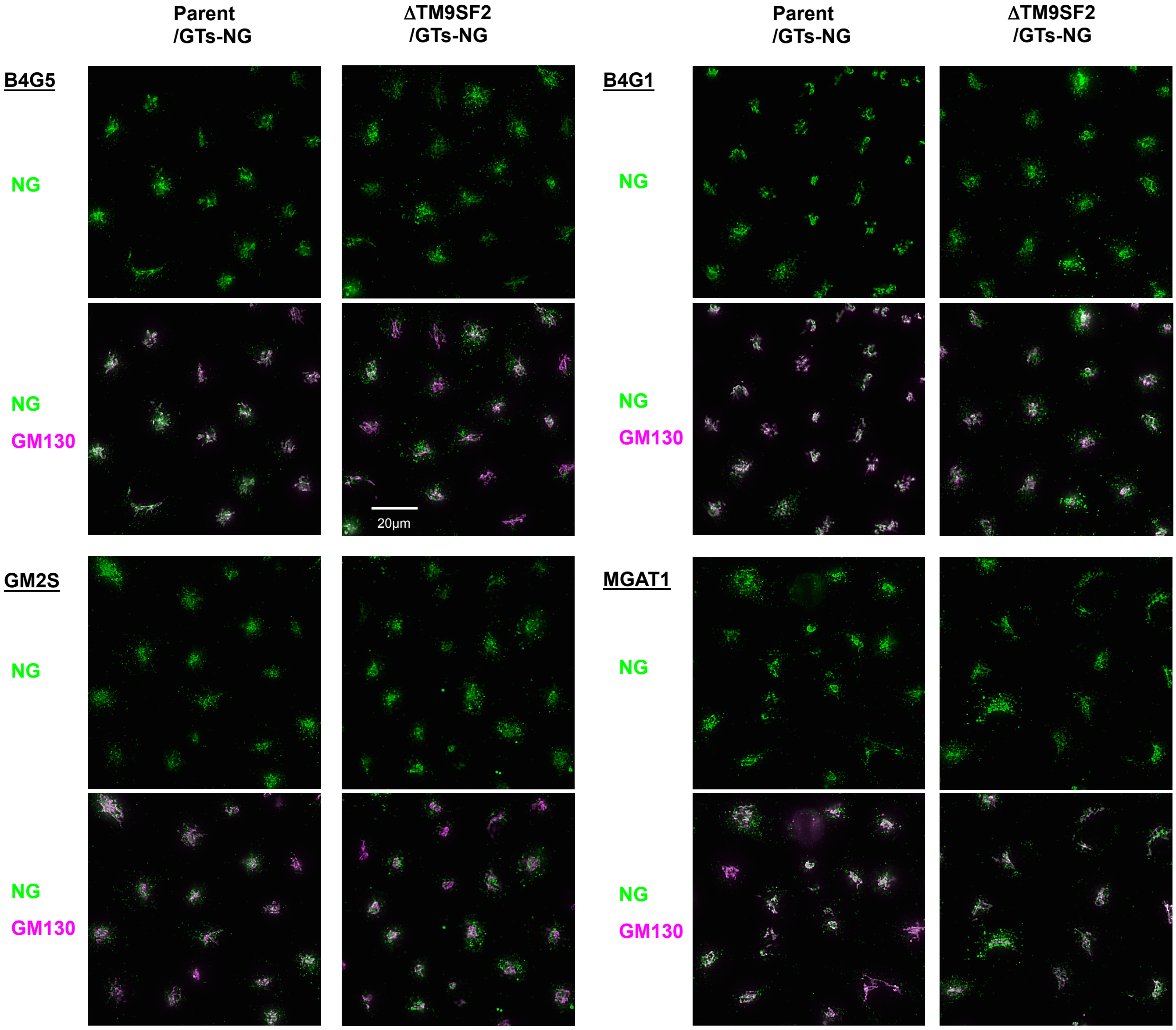

Figure S8. Intracellular Distribution of Glycosyltransferases in ΔTM9SF2 Cells, Related to Figure 7. Parent cells and ΔTM9SF2 cells, expressing Gb3S-NG, B4GalT5 (B4G5)-NG, GM2 synthase (GM2S)-NG, B4GalT1 (B4G1)-NG, and MGAT1-NG, were stained with anti-GM130. Scale bars, 20 µm.

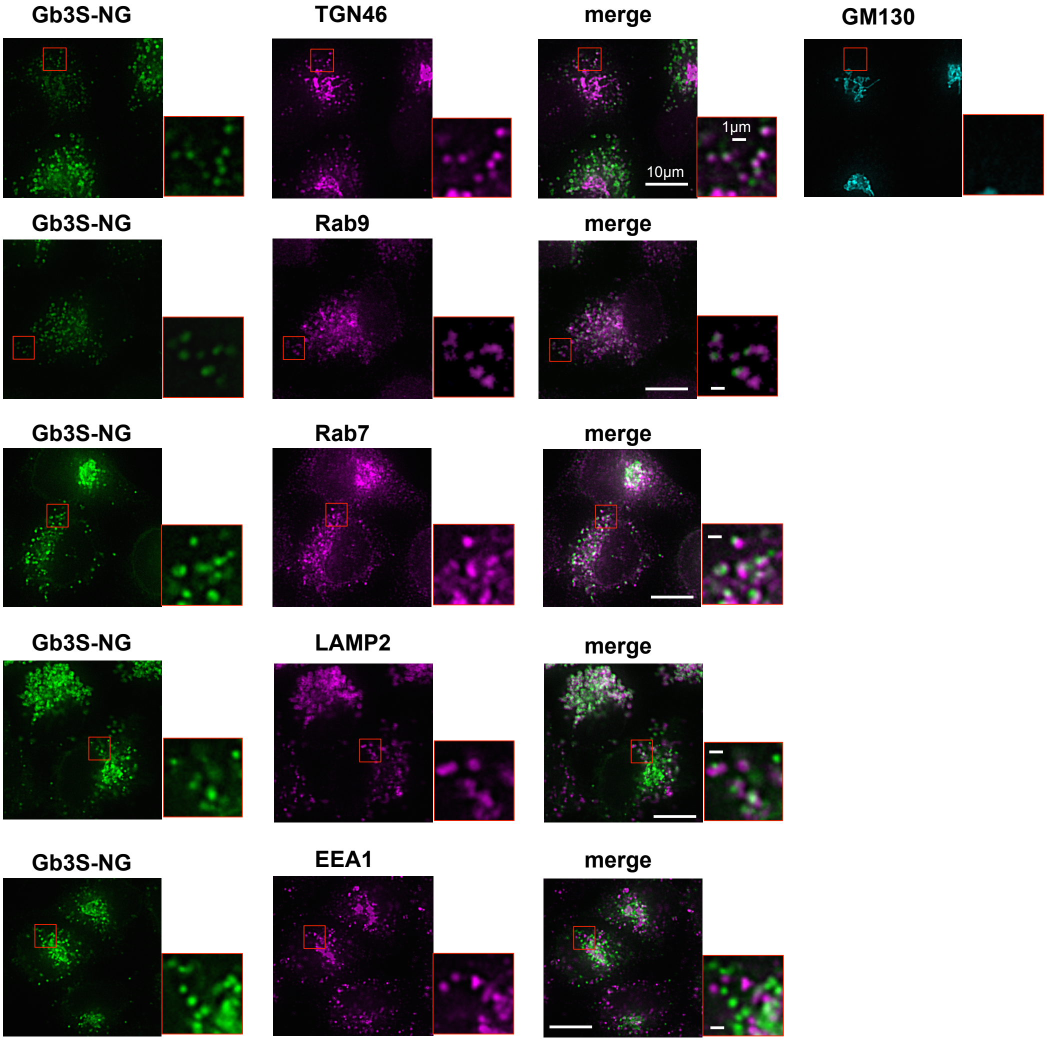

Figure S9. Colocalization of Dispersed Punctate Structures with TGN46, Related to Figure 7. ΔTM9SF2/Gb3S-NG cells were stained with the indicated antibodies (anti-GM130 (cis/medial-Golgi), anti-TGN46 (TGN), anti-Rab9 (late endosome), anti-Rab7 (late endosome), anti-LAMP2 (lysosome and late endosome), and anti-EEA1 (early endosome)). Scale bars, 10 µm and 1 µm.

TRANSPARENT METHODS

Cell Culture, Antibodies, and Reagents

The HeLa-mCAT#8 clone, which expresses mouse cationic amino acid transporter 1 (which serves as the mouse ecotropic retroviral receptor) (Yamaji et al., 2010), and its KO mutants and transfectants were maintained in Dulbecco's modified Eagle's medium (DMEM) containing 10% heat-inactivated fetal bovine serum (FBS) and 4.5 g/L glucose. 293FT cells (ThermoFisher, Rockford, USA) used for lentivirus production were maintained in DMEM containing 10% FBS with non-essential amino acids and sodium pyruvate. Plat-E cells (Morita S et al., 2000) for retrovirus production were maintained in DMEM containing 10% FBS with 1 μg/ml puromycin and 10 μg/ml blasticidin. Sphingolipid-remodeled cells including Gb3 synthase knockdown cells (shGb3S), UGCG KO cells, B4GalT KO cells, and ST3Gal5-overexpressing cells were constructed and described previously (Yamaji et al., 2010, Yamaji et al, 2014).

Purchased antibodies (Abs) were as follows: rabbit anti-LAPTM4A Abs (N-term) (Abgent, SanDiego, USA), rabbit anti-TM9SF2 Abs (ThermoFisher), rabbit anti-Gb3 synthase Abs and rat anti-HA IgG (Sigma-Aldrich, St. Louis, MO), mouse anti-GM130 IgG and mouse anti-EEA1 IgG (BD Transduction Laboratories, San Diego, CA), sheep anti-TGN46 Abs (Serotech, Kidlington, UK), mouse anti-Rab9 IgG (Merck Millipore, Darmstadt, Germany), rabbit anti-Rab7 Abs and rabbit anti-mNeonGreen Abs (Cell Signaling, Danvers, USA), mouse anti-LAMP2 IgG (Santa Cruz Biotechnology, Dallas, USA), mouse anti-mNeonGreen IgG (Chromotek, Planegg-Martinsried, Germany). Chicken anti-VAP-A Abs were raised against the recombinant cytosolic domain of human VAP-A and affinity-purified as described previously (Yamaji et al., 2010). Alexa-conjugated secondary antibodies were purchased from ThermoFisher, except Alexa-594 donkey anti-sheep $F(ab')_2$ fragment, which was purchased from Jackson ImmunoResearch (West Grove, USA).

3-(4,5-Dimethylthiazoyl-2-yl)-2,5-diphenyltetrazolium bromide (MTT) and puromycin were purchased from Sigma-Aldrich. Thin-layer chromatograpy (TLC), high-performance thin-layer chromatography (HPTLC) plates (Silica Gel 60) and Uridine 5' diphosphate galactose (UDP-Gal) were purchased from Merck (Darmstadt, Germany). D-[1-^{14}C]Galactose (56 mCi/mmol) was purchased from GE Healthcare (Buckinghamshire, UK). UDP-[6-^{3}H]Gal was purchased from American Radiolabeled Chemicals (St. Louis, USA). L-[U-^{14}C]Serine (174 mCi/mmol) was purchased from Moravek (Brea, USA). Geneticin was purchased from Nacalai Tesque (Kyoto, Japan). Blasticidin-S was purchased from Kaken Pharmaceutical (Tokyo, Japan). Lipofectamine LTX reagent was purchased from

Thermo Fisher. Polyethylenimine Max (PEI-Max) was purchased from Polysciences Inc (Warrington, USA). Shiga toxin 1 (STx1) derived from *E. coli* O157:H7 was a kind gift from Dr. Kiyotaka Nishikawa (Doshisya University, Kyoto, Japan) (Watanabe et al., 2004). Preparation of fluorescent STx1 B subunit (Alexa555-STx1B) was conducted as described previously (Yamaji et al., 2010). The human Genome-scale CRISPR Knock-Out (GeCKO) v2.0 library in the lentiGuide-Puro plasmid (65386 single-guide RNAs (sgRNAs) in library A and 58031 sgRNAs in library B) and the lentiCAS9-Blast plasmid (two vector lentiviral GeCKO system) were obtained from Addgene (Sanjana et al., 2014). Primers used in this study are described below.

Isolation of Cas9-expressing HeLa cell clone for CRISPR screen

293FT cells were transfected with a lentiCAS9-Blast plasmid and ViraPower packaging plasmids (Thermo Fisher) to produce lentivirus for CAS9 expression (CAS9-lentivirus). Subsequently, HeLa mCAT#8 cells were infected with the CAS9-lentivirus, and the CAS9-expresing cells were grown in the presence of 7.5 μg/ml blasticidin. The clone with the highest genome-editing efficiency was selected as the parent cell clone (HeLa CAS9#W7) for the CRISPR screen.

Production of lentiviral CRISPR libraries

For amplification of the GeCKO v2.0 library plasmids, 300 ng of the library A and B plasmids were separately transformed into ElectroTen-Blue Electroporation-Competent cells (Agilent, Santa Clara, USA) using a Gene Pulser Xcell (BIO-RAD, Hercules, USA), and the plasmids were purified from approximately 5–10 × 10^7 colonies of transformed *E.coli* using an Endotoxin-free Plasmid DNA Extraction Maxi Kit (Favorgen, Ping-Tung, Taiwan). Two independent lentiviral pools (#1 and #2) of the library were produced. Briefly, approximately 1 × 10^8 (#1) and 7 × 10^7 (#2) 293FT cells were transfected with 48 μg (#1) and 30 μg (#2) of the library plasmids and ViraPower packaging plasmids (Thermo Fisher) using PEI Max (#1) and Lipofectamine LTX with Plus reagent (#2). After 24 hours, media was changed and the cells were cultured for 24 additional hours. Subsequently, culture media containing secreted lentiviruses was filtered using a 0.45 μm bottle top filter and frozen as lentiviral pools (A-1, A-2, B-1, B-2).

Preparation of sgRNA-expressing cell libraries

HeLa CAS9#W7 cells (2 × 10^7 cells) were infected with each lentivirus pool at a low MOI (about 0.2). Twenty-four hours after transduction, cells were selected with 1 μg/ml puromycin for three days and cultured for an additional five days to prepare sgRNA-expressing HeLa cell libraries.

CRISPR screen for STx1 treatment

2.4 × 10^7 sgRNA-expressing cells from each cell library (A-1, A-2, B-1, B-2) were plated 24 hours prior to treatment with 50 pg/ml STx1. Three days after the STx1 treatment, cells were cultured in the absence of Stx1 for 8 days. Surviving cells were then re-plated and treated with 50 pg/ml STx1 again. Two days after treatment, cells were trypsinized and frozen as cell pellets. For untreated controls, 1.2 × 10^7 sgRNA-expressing cells in each cell library were cultured for the same period as STx1-treated cells with several passages, such that a minimum of 1.2 × 10^7 cells was present in each passage.

Genomic DNA sequencing

Analysis of genome-integrated sgRNAs was based on new generation sequencing using MiSeq (Illumina, San Diego, USA). Genomic DNA from frozen cells was purified using the conventional phenol-chloroform method. Briefly, cell pellets were re-suspended in 5ml QIAGEN Buffer P1 with 0.5% SDS, and were sonicated to shear DNA. Subsequently, a phenol/chloroform extraction, a chloroform extraction, and DNA precipitation with isopropanol were performed. Amplification of the genome-integrated sgRNA sequences by PCR was performed as follows, based on a previous report (Shalem et al., 2014). For the first PCR, 100 μg genomic DNA from untreated cells or more than one third of the total amount of isolated genomic DNA from STx1-treated cells were used as PCR templates. For each sample (A-1, A-2, B-1, B-2), nine separate 100 μl reactions were performed using PrimeStar GXL DNA polymerase (Takara, Otsu, Japan) and the following primers (9 forward primers and 1 reverse primer):

(Fw) 1stY1R1s0-8: CTACACGACGCTCTTCCGATCT (0-8 bp random sequence for increasing library complexity) TCTTGTGGAAAGGACGAAACACCG

(Rv) 1stY2as: GCCACTTTTTCAAGTTGATAACGGACTAG

Amplification was carried out with 20 cycles. One (1) μl from these nine separate first PCR products was respectively used as a template for the second PCR. For each sample, nine separate 20 μl PCR reactions were performed using the following primers:

(Fw) 2nd P5R1s: AATGATACGGCGACCACCGAGATCTACA**CTCTTTCCCTACACGACGCTC TTCCGATCT**

(Rv) 2nd P7Y2as: CAAGCAGAAGACGGCATACGAGAT (CC (A-1), or TT (A-2), or AA (B-1), or GG (B-2) as barcodes for multiplexing of different samples) GCCACTTTTTCAAGTTGATAACGGACTAG

Underlines indicate Illumina adaptor sequences (P5 and P7 respectively), with bold letters indicating the sequence

primer site for MiSeq sequence analysis. Amplification was carried out with 10 cycles. The resulting nine amplicons in each sample were mixed and gel extracted using SYBR Gold (ThermoFisher). The extracted DNA was then quantified using a Quantus fluorometer (Promega, Madison, USA) as well as running an agarose gel with 100 bp quantifiable DNA Ladder (NEB, Ipswich, USA), and equal amounts of each sample (A-1, A-2, B-1, B-2) were mixed. The DNA concentration of the mixture was adjusted for sequencing analysis. PhiX Control Kit v3 (Illumina) was added to the sample at approximately 20% concentration. MiSeq Reagent Kit v3 (Illumina) was used for MiSeq sequencing.

Data processing and analysis

To perform demultiplexing of fastq sequence data, total raw read sequences were divided into each sample with barcode sequences of "AA", "CC", "GG" and "TT" using an in-house program. The adapter sequences were removed using a skewer program (version 0.1.126) (Jiang et al., 2014) with the following parameters: minimum read length = 10 mer, maximum read length = 30 mer, lowest mean quality value = 19 sanger quality score. To extract high-quality sgRNA sequences, sequences with a Phread quality score less than 20 were excluded using the "split_libraries_fastq.py" (version 1.9.1) function of the QIIME program (Caporaso et al., 2010). The numbers of the sgRNA sequences were calculated with "sort" and "uniq" of the unix command program, followed by normalization with the following formula; normalized reads per sgRNA = reads per sgRNA / total reads for all sgRNAs in sample x 10^7 (Data S1). Fold enrichment was calculated using the following formula: Fold enrichment = normalized reads in STx-treated sample / normalized reads in untreated sample. When the normalized reads in the untreated sample was 0, fold enrichment was calculated by setting 0 to 1. First, identification of essential genes, which were closely related to STx1 interaction, was performed using the MAGeCK program (version 0.5.7) (Li et al., 2014) to analyze normalized sgRNA count data (Data S3). In this program, 640 genes contained at least one significantly different sgRNA. For stricter selection of hit sgRNAs, the sgRNAs representing more than 1-fold enrichment in both independent cell libraries (A-1 and A-2, or B-1 and B-2) were selected as STx resistance sgRNA candidates (Data S2), and fold enrichment of these candidates were graphed in Figure 1A. Note that the selected sgRNAs were all statistically significantly enriched, which was demonstrated using the MAGeCK program (Data S1 and S2).

Synthesis of CRISPR plasmids

For selection with puromycin to remove untransfected cells in genome-editing, the pSELECT-CRISPR-CAS9 plasmid (Ogawa et al.,2018) was used. The plasmid was cleaved with BsmBI, and a 20-mer guide sequence was

ligated into the site. The sequences of the 20-mer guide sequence were confirmed using an ABI3100 sequencer. The sgRNA sequences used in this study were described below.

Construction of CRISPR KO cell lines

On day 0, HeLa-mCAT8 cells (1.5×10^5 cells/well in 12-well plates) were cultured overnight. On day 1, a CRISPR plasmid was mixed with X-tremeGENE HP (Roche Diagnostics) (in 12-well plates, 1 µg of plasmid and 2 µl X-tremeGENE HP were mixed in 100 ml Opti-MEM), and the mixture was then added to the cells. On day 2, the cells were transferred to 6-well plates and cultured at 37°C with puromycin at 5 µg/ml, which is higher than the usual concentration, in order to concentrate cells with higher sgRNA expression. This step excludes the untransfected cells. On day 5, culture medium was changed to puromycin-free medium, and the cells were subcultured for 3 days. CRISPR-treated HeLa cells were used for STx treatment, and the cell viability assay was conducted as described below (Figure 1B), harvested for indel analysis, or diluted to isolate gene-disrupted clones. To construct HA-tagged knock-in cells by homologous recombination using the CRISPR/CAS system, single-stranded oligonucleotides of C-terminus of Gb3S with HA-tag were transfected together with a CRISPR plasmid targeting Gb3 synthase. The sequence of the single-stranded oligonucleotides is described below.

Indel analysis

Indel analysis was performed as previously described (Yamaji and Hanada, 2014). Briefly, trypsinized cells were heated in TE buffer followed by vortexing to use as a template of genomic PCR. PCR was performed with PrimeSTAR GXL, and blunt-end PCR products were then cloned with a Zero Blunt TOPO PCR Cloning Kit (Invitrogen). After *E.coli* transformation, colony direct PCR or plasmid purification was performed to use as a template for sequence analysis. DNA sequences were determined using an ABI3100 Genetic Analyzer (Applied Biosystems). KO cell clones of *LAPTM4A*, *TM9SF2*, *SPTSSA*, *ACACA*, *TMEM165*, and *A4GalT* (*Gb3S*) were isolated. A resolvase-based mutation assay using the Guide-it Mutation Detection Kit was performed according to the manufacture's instructions (Takara).

RNA isolation, RT-PCR, and real-time PCR

Total RNA was isolated using the TRIzol Reagent per manufacturer's instructions (Thermo Fisher). RT-PCR was performed using the ReverTra Ace qPCR RT Master Mix (ReverTra Ace, Toyobo) per manufacturer's instructions, including a DNase I treatment step. For real-time PCR, the LightCycler 96 system with LightCycler-FastStart DNA

master SYBR Green I kit (Roche) was used according to the manufacturer's protocol (Yamaji et al., 2010).

cDNA cloning and vector construction

Human *TM9SF1-4*, *LAPTM4A*, *LAPTM4B*, *TMEM165*, and *Gb3 synthase* (*Gb3S*) cDNAs were amplified by PCR (template: *TM9SF1* and *TM9SF3* from thyroid cDNA (Thermo Fisher), *TM9SF4* from placenta cDNA, *Gb3S* from brain cDNA, others from HeLa mCAT#8 cDNA). Amplified DNAs were digested with restriction enzymes and inserted into the vectors as described below. moxNeonGreen (C149T) was prepared from mNeonGreen (Allele Biotechnology, San Diego, USA) by PCR-based mutagenesis to prevent non-native disulphide bonds in the lumen of the Golgi (Shaner et al., 2013, Costantini et al., 2015). Gb3S was fused with moxNeonGreen at the C-terminus to prepare Gb3S-moxNeonGreen fusion protein (Gb3S-NG). The sequences of LAPTM4A and LAPTM4B were aligned using EMBL-EBI EMBOSS Water (https://www.ebi.ac.uk/Tools/psa/emboss_water/). spHA-TM9SF2 was constructed by PCR and Gibson assembly (NEB). pCXN$_2$-GRINA TM4-6-HA plasmid (for use as a negative control) was constructed and described previously (Yamaji et. al., 2010).

Preparation of plasmid-based stable transfectants

pCXN$_2$-TM9SF2 plasmids were linearized and transfected into *TM9SF2*-KO cells using X-tremeGENE HP. The cells were then subjected to geneticin selection at a concentration of 800 μg/ml. Colonies were isolated by limiting dilution. A clone expressing TM9SF2 proteins was selected (ΔTM9SF2/TM9SF2).

Retroviral infection and preparation of stable transfectants

Preparation of retroviruses and infection of HeLa-mCAT#8-based cells were performed using the Plat-E system, as described previously (Morita S et al., 2000). When pMXs-IP-based (Gb3S-NG, B4G5-NG, GM2S-NG, B4G1-NG, MGAT1-NG) and pMXs-IB-based (LAPTM4A, its mutants, LAPTM4B, TMEM165, and Gb3S-NG) retroviruses were used, the concentrations of puromycin and blasticidin-S for selection were 2 μg/ml and 7.5 μg/ml, respectively. Established cells were as follows: ΔLAPTM4A/LAPTM4A, ΔLAPTM4A/LAPTM4A-HA, ΔLAPTM4A/HA-LAPTM4A, ΔLAPTM4A/LAPTM4AΔC-HA ΔLAPTM4A/LAPTM4B-HA, ΔTMEM165/TMEM165, Parent/Gb3S-NG, ΔLAPTM4A/Gb3S-NG, ΔLAPTM4A/LAPTM4A-HA/Gb3S-NG, ΔLAPTM4A/HA-LAPTM4A/Gb3S-NG, ΔLAPTM4A/LAPTM4AΔC-HA/Gb3S-NG, ΔTM9SF2/Gb3S-NG, ΔTM9SF2/TM9SF2/Gb3S-NG, Parent/Gb3S, ΔLAPTM4A/Gb3S, ΔLAPTM4A/LAPTM4A-HA/Gb3S, ΔLAPTM4A/HA-LAPTM4A/Gb3S, and ΔLAPTM4A/LAPTM4AΔC-HA/Gb3S. Gb3S-NG-expressing cells were

further cloned, and representative clones, including Parent/Gb3S-NG#1, #2, Parent/ Gb3S-NG#IB1, ΔTM9SF2/Gb3S-NG#1, #2, and ΔTM9SF2/TM9SF2/Gb3S-NG#1, were used. Mean fluorescence intensities are indicated in the images. HeLa shGb3S cells were constructed and described previously (Yamaji et al., 2010).

Immunofluorescence microscopy

Immunostaining was performed as described previously (Kawano et al., 2006), and specimens were visualized with a wide-field fluorescence microscope, BZ-X700 (Keyence, Osaka, Japan) equipped with a Plan Apo VC 60x1.20 WI (water immersion) objective. Haze reduction function (condition 2), which applies a no-neighbor deconvolution algorithm to the captured image, was used to eliminate fluorescence blurring caused by scattered light and capture clear images with high contrast.

Lysate preparation and Western blot analysis

Two methods were used to prepare lysates as follows. Method 1: Cells were sonicated in sonication buffer (10 mM Hepes/NaOH (pH7.4) 1 mM EDTA, 0.25 M sucrose, protease inhibitor cocktail) and subsequently mixed with Laemmli sodium dodecyl sulfate (SDS) sample buffer. This method was used for the detection of Gb3 synthase. Method 2: Cells were sonicated as in Method 1. For the detection of LAPTM4A, the lysates were ultracentrifuged at 100,000g for 1 hr at 4°C to isolate membrane fractions, followed by suspension in sonication buffer. Then, lysates were mixed with 4 volumes of urea-containing buffer (50 mM Tris/HCl pH 8.8, 7 M urea, 2 M thiourea, 2% CHAPS, 2% Triton X-100, 33 mM DTT, protease inhibitor cocktail), and incubated for 1 hr at 37°C. Proteins were then alkylated with 100 mM iodoacetoamide to prevent re-oxidation of SH residues. Lithium dodecyl sulfate was added to samples at 2%. This method was used for the detection of TM9SF2 and LAPTM4A, which are multispanning membrane proteins. Protein concentrations were determined using the Pierce BCA protein assay kit using BSA as a standard. Proteins were resolved by SDS-PAGE, transferred to PVDF membranes using the wet transfer method, and probed with specified antibodies. Antigen signals were detected using SuperSignal West Femto Maximum Sensitivity Substrate (ThermoFisher Scientific) or Chemi-Lumi One L (Nacalai, Kyoto, Japan) and exposed to an X-ray film. To detect TM9SF2, urea-containing polyacrylamide gels were used, as the predicted molecular size of TM9SF2 band deviated from the molecular marker (Bio-rad, Hercules, USA) when urea was not included, suggesting urea was required for protein denaturation.

Immunoprecipitation analysis

Cells were lysed with Lysis buffer (50mM Tris/HCl pH8.0, 150mM NaCl, 1mM EDTA, 1% Triton). After

centrifugation, the cell lysate supernatants were incubated with anti-HA agarose beads (Sigma) or mNeonGreen magnetic agarose beads (Chromotek). After washing with Lysis buffer, bound proteins were eluted with SDS sample buffer.

Metabolic labeling of glycolipids and TLC analysis

Metabolic labeling experiments using L-[U-^{14}C]serine and D-[1-^{14}C]galactose including mild alkaline methanolysis were performed as described previously (Yamaji et al., 2016). Cells (3 × 10^5/well in a 6-well plate) were cultured overnight at 37°C, and cells were then incubated with 22.2 kBq of L-[U-^{14}C]serine or 7.4 kBq of D-[1-^{14}C]galactose in Opti-MEM with 1% Neutridoma-SP (Roche) for 16 h. Cells were lysed with 0.1% sodium dodecyl sulfate (SDS), and lysates containing the same amount of protein were then used for lipid extraction following the method of Bligh and Dyer (Bligh and Dyer 1959). For alkali-methanolysis to remove glycerolipids, dried lipids were hydrolyzed with 0.1N KOH in methanol for 1 hr at 40°C. After neutralization with 0.1N HCl, the methanol layer was washed with *n*-hexane twice, and the lipids were extracted using the method of Bligh and Dyer. The lower fractions collected were dried under an N_2 gas stream. Separation of lipids by TLC was performed using two methods as follows. Method 1: A TLC60 plate (20 cm × 20 cm), developing solvent: methyl acetate/n-propanol/chloroform/methanol/0.25% KCl = 50/50/50/20/18 (Yamaji et al., 2014), or Method 2: A HPTLC60 plate (10 cm × 20 cm), developing solvent: chloroform/methanol/0.25% $CaCl_2$ = 65/35/8 (Yamaji et al., 2010). Method 1 was used in Figures 2E, S1D, and S1E, and Method 2 was used in Figures 3B, S1F, and S1G. The radioactive lipids on TLC plates were visualized, and the intensity of each band was quantified using a Typhoon FLA 7000 (GE Healthcare, Buckinghamshire, UK). To compare relative amounts of the lipids, the band intensity of each GSL in the parent cells was considered to be 100%.

Measurement of *in vitro* Gb3 synthase activity

To measure Gb3 synthase activity *in vitro,* 5 × 10^6 cells/plate in a 15cm plate were cultured overnight at 37°C, and cells were then scraped and sonicated in sonication buffer (10 mM Hepes/NaOH (pH7.4), 1 mM EDTA, 15 mM $MnCl_2$, 0.5% Triton X-100, 0.25 M sucrose, protease inhibitor cocktail (Roche)). The lysates were adjusted by total protein concentration, which was used as an enzyme source. Twenty-five micrograms of LacCer (or no lipid) and 300 μg Triton X-100 were dissolved in chloroform, which was removed from the mixture using an N_2 gas stream. Dried lipids were sonicated in 90 μl reaction buffer (20 mM MES/NaOH (pH 6.4), 15 mM $MnCl_2$, 6.25 μM UDP-galactose) and substrates were then incubated with 25 pmol (0.5 μCi) of UDP-[6-^{3}H]galactose and 10 μl of the prepared lysates for 1 hr at 37°C. Lipids in the reaction mixture were extracted using the method of Bligh and Dyer.

Separation of the lipids by TLC was performed through Method 2 described above. Visualization and analysis of the labeled lipids were performed as above. The value of the band intensity with LacCer minus the band intensity without LacCer was regarded as the relative Gb3 synthase activity. To compare relative Gb3 synthase activity, the value in the parent cells was considered to be 100%.

FACS analysis

Non-confluent cells were trypsinized and washed with culture medium and wash buffer (1% BSA) in PBS at 4°C. Cells were incubated with 10 μg/ml Alexa-555 Stx1 B subunits for 45min on ice. After washing with wash buffer once, cells were analyzed using a FACSCalibur (BD Biosciences, Franklin Lakes, USA). To determine the effect of transiently expressed proteins on the expression of STx receptors, TM9SF2 KO cells (2.5×10^4 cells/well in a 12-well plate) were co-transfected with 0.5 μg $pCXN_2$ plasmids containing the target genes (TM9SFs and TM9SF2 mutants) and 0.05 μg EGFP-N3 (Clontech, Mountain View, USA). After two days of transfection, cells were subjected to FACS analysis. Spillover of EGFP fluorescence in the FL2 channel, and spillover of Alexa-555 in the FL1 channel were electronically compensated. After gating out debris and cell aggregates by FSC/SSC, the percentage of StxR upper cells in EGFP-positive cells was calculated by (cell number in upper right) / (cell number in upper and lower right) x 100.

STx treatment and cell viability assay

To treat with STx1, cells ($1\text{-}1.5 \times 10^4$ cells/ml in 12-well or 24-well plates) were cultured overnight at 37°C, and then treated with STx1 at the indicated concentrations for three days. An MTT assay was then performed as described previously to assess cell viability (Yamaji et al., 2010).

Statistical analysis

A two-tailed unpaired Student's *t*-test was used for statistical analysis, with $p < 0.05$ considered to be statistically significant. For multiple comparisons, the Student's *t*-test with Bonferroni correction was used, with $p < 0.01$ (0.05 divided by 5) considered to be statistically significant in five comparisons (Figure 2F, 3A, and 3C) and $p < 0.0083$ (0.05 divided by 6) considered to be statistically significant in six comparisons (Figure 2C).

Primers used in this study

<u>Primers for indel analysis</u>

hTM9SF2 5UTRs: CCTTGTAGTCGTCTCCGAGAC

hTM9SF2 Ex1as: TCTTCGTCGCAGAAGTTGACGG

hLAPTM4A 5UTRs: CGTGAAACAGCCGTTTGAGTTTGG

hLAPTM4A Ex1as: ATGTACCAGGTCCCCAGGATG

hSPTSSA 5UTRs: GACAGACTGACGTGTGAGCTG

hSPTSSA Int1as: GGATCTCAAGAGTTCTCGTCTCC

hACACA-Ex9s: GTTCTTATTGCTAACAATGGCATTGCAGC

hACACA-Int9as: CCTACTTAAAGGCTGTGCTGTTCCATG

hTMEM165 5UTRs: TGTTCGGGGTCGAGGCTTC

hTMEM165 Ex1as: GGTTCTTTGTTCCGGTGGCTAAG

hGb3S-Ex3s: ACCAGCCGGTTCCTGCTGGAAG

hGb3S-Ex3as: GGTTATAGAGCTGCCCTTTCTCC

Primers for constructing expression vectors (Underlines are indicative of restriction enzyme cutting sites)

hTM9SF2 BglII-ATGs: ACCAGATCTCCCGGGTATCATGAGCGCGAGGCTGC

hTM9SF2-XhoI-STOPas: ACCCTCGAGTCAGTCAACCTTCACCACACTG

hTM9SF2 XhoI-ENDas: ACCCTCGAGGTCAACCTTCACCACACTGTATATTTTG

hTM9SF1-SacI-ATGs: ACCGAGCTCAGGATGACAGTCGTAGGGAAC

hTM9SF1-XhoI-STOPas: ACCCTCGAGAACTCAGTCCATCTTGAGGTTAACATAG

hTM9SF3-SacI-ATGs: ACCGAGCTCAGGATGAGGCCGCTGCCT

hTM9SF3-XhoI-STOPas: ACCCTCGAGGGGTCTCTAGTCAATTTTCACATTAG

hTM9SF4-RI-ATGs: ACCGAATTCAAGATGGCGACGGCGATGGATTG

hTM9SF4-Xho-STOPas: ACCCTCGAGTCAGTCTATCTTCACAGCAGCATAGATC

hTM9SF2 SVN STOPas: ACCCTCGAGTCAATTAACAGACACCACACTGTATATTTTGGTAAC

hTM9SF2 AVA STOPas: ACCCTCGAGTCAAGCAACAGCCACCACACTGTATATTTTGGTAAC

hTM9SF2 CΔ3 STOPas: ACCCTCGAGTCACACCACACTGTATATTTTGGTAAC

hLAPTM4A BamHI-ATGs: ACCGGATCCACGATGGTGTCCATGAGTTTCAAGC

hLAPTM4A XhoI-STOPas: ACCCTCGAGTCAGGCAGGTAAGTAAGGAGGTG

hLAPTM4A XhoI-ENDas: ACCCTCGAGGGCAGGTAAGTAAGGAGGTGGTGG

hLAPTM4A XhoI-NCY(ΔC)as: ACCCTCGAGATAGCAGTTCCAAACACAGTTAATTAGATAAGCC

hLAPTM4B BamHI-ATGs: ACCGGATCCGCGATGACGTCACGGACTCGGGTC

hLAPTM4B XhoI-ENDas: ACCCTCGAGGGCAGACACGTAAGGTGGC

hTMEM165 BamHI-ATGs: ACCGGATCCTGGTGCTGACTGCTCCCTAAG

hTMEM165 XhoI-STOPas: ACCCTCGAGTTAAAAACCAGAATCAGGGCTTATAAATAGTGC

Gb3S XhoI-ATGs: GCGCTCGAGATACCATGTCCAAGCCCCCCG

Gb3S HindIII-ENDas: GCGAAGCTTCAAGTACATTTTCATGGCCTCGTGCGTC

mNeonGreenVec5side-s: CTGGTTTAGTGAACCGTCAGATCC

mNeonGreenVec3side-as: CCTCTACAAATGTGGTATGGCTG

mNeonGreenCTs: GCTGCGGACTGG**ACC**AGGTCGAAGAAGACT (bold: mutation site)

mNeonGreenCTas: CTTCTTCGACCT**GGT**CCAGTCCGCAGCGGT (bold: mutation site)

hTM9SF2 N-HAas (spHA-TM9SF2): GTCCGGGACGTCATATGGGTACAGGCCGGGCAGGTAGAAAGC (underlined: HA-tag sequence)

hTM9SF2 N-HAs (spHA-TM9SF2): TATGACGTCCCGGACTACGCGCCCGTCAACTTCTGCGA (underlined: HA-tag sequence)

Primers for RT-PCR and real-time PCR

TM9SF1: hTM9SF1-SacI-ATGs and hTM9SF1-XhoI-STOPas described above

TM9SF3: hTM9SF3-SacI-ATGs and hTM9SF3-XhoI-STOPas described above

TM9SF4: hTM9SF4-RI-ATGs and hTM9SF4-Xho-STOPas described above

Gb3S s: GGCAACATCTTCTTCCTGGAGACTTC

Gb3S as: CGAACTTCCACATGAGTGCGATCC

GAPDH s: GAGTCAACGGATTTGGTCGT

GAPDH as: TTGATTTTGGAGGGATCTCG

sgRNA target sequences

Figure 1B and Figure S1A-C

SPTSSA: ACTGAACACCGTCCGCTCCC

A4GalT (Gb3S): CCCGCTGCCTTGGGGCGCCC

LAPTM4A (v3): CCAGGATGATCGTCCCGGTG

ACACA: GAAGACCTTAAAGCCAATGC

COG4: TCTAGGGATTGCCCGCATTG

TM9SF2 (v2): TGTGAACAGACTTGATTCAG

VPS54: TACTTGCTCCAGATCTGTCC

CAMLG: ACAGCGCATCAACCGGATCA

GOSR1: TCAATACCTACTTTCGAAGG

UNC50: AATCTATGAGTACAACCCAA

GET4: ACGAGGCGCACCAGATGTAC

PI4KB: GTATGAGCCAGCTGTTCCGAA

TMEM165: GCAGCCGGGGCGCCGATGCG

NBAS: ATCATCACGAAAGCAATTCG

ZDHHC17: ATGGAATGACGCCTTTAATG

PTAR1: TAACCGGAGTCCCATAGTCC

AAED1: AATGTCACCCTTATAGTGAT

TRAPPC12: GCCCGCTCTTGCCGTAGCCC

ARL1: ATCAGAGTTAGTTGCCATGT

FURIN: TCACTCCTCGATGCCAGAAG

STX16: TTAGATCCAGAAGCAGCGAT

Figure 2A

TM9SF2 (v3): AAACAACTATCATGAGCGCG
LAPTM4A (v4): GCGGTTCCGCTTGAAACTCA

Figure S2E

A4GalT (C): CCATGAAAATGTACTTGTGA
Oligonucleotide for homologous recombination:
CTGGCCCAGCTGCATGCCCGCTACTGCCCCACGACGCACGAGGCCATGAAAATGTACTTGTACCCATACGATGTTCCAGATTACGCT**TGA**GGGGCCCGCCAGGTCACCTCCCCAACCTGCTCCTGATGGGGCACTGGGCCGCCCTTC (underline: HA-tag, bold: STOP codon)

Figure S5

TM9SF1: ATACAGAATAACAGGGTCGC
TM9SF3: CGTGCTCGTCCGCCCGGGTC
TM9SF4: TGATTTCTACGGGATCGTTC

Plasmids constructed in this study

$pCXN_2$-hTM9SF2 and spHA-TM9SF2 (and its mutants): cDNA (Blunt end-XhoI), plasmid (EcoRV-XhoI)
$pCXN_2$-hTM9SF2-HA: cDNA (Blunt end-XhoI), HA-tag (XhoI-NotI), plasmid (EcoRV-NotI),
$pCXN_2$-hTM9SF1 and hTM9SF3: cDNA (SacI-XhoI), plasmid (SacI-XhoI)
$pCXN_2$-hTM9SF4: cDNA (EcoRI-XhoI), plasmid (EcoRI-XhoI)
pMXs-IB-hLAPTM4A and hTMEM165: cDNA (BamHII-XhoI), plasmid (BamHI-XhoI)
pMXs-IB-hLAPTM4A-HA (and its mutant-HAs) and LAPTM4B-HA: cDNA (BamHII-XhoI), HA-tag (XhoI-NotI), plasmid (BamHII-NotI)
pMXs-IB-HA-hLAPTM4A: HA-tag (BglII-BamHI), cDNA (BamHI-XhoI), plasmid (BamHI-XhoI)
pMXs-IP-Gb3S-moxNeonGreen (NG): Gb3S (XhoI-HindIII), NG (HindIII-NotI), plasmid (XhoI-NotI)
pMXs-IB-Gb3S-moxNeonGreen (NG): Gb3S (XhoI-HindIII), NG (HindIII-NotI), plasmid (XhoI-NotI)
pMXs-IP-B4G5-moxNeonGreen (NG): B4G5 (EcoRI-HindIII), NG (HindIII-NotI), plasmid (XhoI-NotI)
pMXs-IP-GM2S-moxNeonGreen (NG): GM2S (XhoI-HindIII), NG (HindIII-NotI), plasmid (XhoI-NotI)
pMXs-IP-B4G1-moxNeonGreen (NG): B4G1 (XhoI-HindIII), NG (HindIII-NotI), plasmid (XhoI-NotI)
pMXs-IP-MGAT1-moxNeonGreen (NG): MGAT1 (XhoI-HindIII), NG (HindIII-NotI), plasmid (XhoI-NotI)
pMXs-IP-Gb3S was constructed previously (Yamaji et al. 2010)

SUPPLEMENTAL REFERENCES

Bligh, E.G., and Dyer, W.J. (1959). A rapid method of total lipid extraction and purification. Can. J. Biochem. Physiol. 37, 911-917.

Caporaso, J.G., Kuczynski, J., Stombaugh, J., Bittinger, K., Bushman, F.D., Costello, E.K,, Fierer, N., Peña, A.G., Goodrich, J.K., Gordon, J.I., Huttley, G.A., Kelley, S.T., Knights, D., Koenig, J.E., Ley, R.E., Lozupone, C.A., McDonald, D., Muegge, B.D., Pirrung, M., Reeder, J., Sevinsky, J.R., Turnbaugh, P.J., Walters, W.A., Widmann, J., Yatsunenko, T., Zaneveld, J., and Knight, R. (2010). QIIME allows analysis of high-throughput community sequencing data. Nat. Methods 7, 335-336.

Jiang, H., Lei, R., Ding, S.W., and Zhu, S. (2014). Skewer: a fast and accurate adapter trimmer for next-generation sequencing paired-end reads. BMC Bioinformatics 15, 182.

Kawano, M., Kumagai, K., Nishijima, M., and Hanada, K. (2006). Efficient trafficking of ceramide from the endoplasmic reticulum to the Golgi apparatus requires a VAMP-associated protein-interacting FFAT motif of CERT. J. Biol. Chem. 281, 30279-30288.

Li, W., Xu, H., Xiao, T., Cong, L., Love, M.I., Zhang, F., Irizarry, R.A., Liu, J.S., Brown, M., and Liu, X.S. (2014). MAGeCK enables robust identification of essential genes from genome-scale CRISPR/Cas9 knockout screens. Genome Biol. 15, 554.

Morita, S., Kojima, T., and Kitamura, T. (2000). Plat-E: an efficient and stable system for transient packaging of retroviruses. Gene Ther. 7, 1063-1066.

Ogawa, M., Matsuda, R., Takada, N., Tomokiyo, M., Yamamoto, S., Shizukusihi, S., Yamaji, T., Yoshikawa, Y., Yoshida, M., Tanida, I., Koike, M., Murai, M., Morita, H., Takeyama, H., Ryo, A., Guan, J.L., Yamamoto, M., Inoue, J.I., Yanagawa, T., Fukuda, M., Kawabe, H., and Ohnishi, M. (2018). Molecular mechanisms of Streptococcus pneumoniae-targeted autophagy via pneumolysin, Golgi-resident Rab41, and Nedd4-1-mediated K63-linked ubiquitination. Cell Microbiol. , e12846.

Watanabe, M., Matsuoka, K., Kita, E., Igai, K., Higashi, N., Miyagawa, A., Watanabe, T., Yanoshita, R., Samejima, Y., Terunuma, D., Natori, Y., and Nishikawa, K. (2004). Oral therapeutic agents with highly clustered globotriose

for treatment of Shiga toxigenic Escherichia coli infections. J. Infect. Dis. 189, 360-368.

Yamaji, T., and Hanada, K. (2014). Establishment of HeLa cell mutants deficient in sphingolipid-related genes using TALENs. PLoS One 9, e88124.

Yamaji, T., Horie, A., Tachida, Y., Sakuma, C., Suzuki, Y., Kushi, Y., and Hanada, K. (2016). Role of Intracellular Lipid Logistics in the Preferential Usage of Very Long Chain-Ceramides in Glucosylceramide. Int. J. Mol. Sci. 17, E1761.
